# Synapsin is required to “boost” memory strength for highly salient events

**DOI:** 10.1101/lm.039685.115

**Published:** 2016-01

**Authors:** Jörg Kleber, Yi-Chun Chen, Birgit Michels, Timo Saumweber, Michael Schleyer, Thilo Kähne, Erich Buchner, Bertram Gerber

**Affiliations:** 1 Leibniz Institut für Neurobiologie (LIN), Abteilung Genetik von Lernen und Gedächtnis, 39118 Magdeburg, Germany; 2 Otto von Guericke Universität Magdeburg, Institut für Experimentelle Innere Medizin, 39120 Magdeburg, Germany; 3 Institut für Klinische Neurobiologie, 97078 Würzburg, Germany; 4 Center for Behavioral Brain Sciences (CBBS), Magdeburg, Germany; 5 Otto von Guericke Universität Magdeburg, Institut für Biologie, 39106 Magdeburg, Germany

## Abstract

Synapsin is an evolutionarily conserved presynaptic phosphoprotein. It is encoded by only one gene in the *Drosophila* genome and is expressed throughout the nervous system. It regulates the balance between reserve and releasable vesicles, is required to maintain transmission upon heavy demand, and is essential for proper memory function at the behavioral level. Task-relevant sensorimotor functions, however, remain intact in the absence of Synapsin. Using an odor–sugar reward associative learning paradigm in larval *Drosophila*, we show that memory scores in mutants lacking Synapsin (*syn*^*97*^) are lower than in wild-type animals only when more salient, higher concentrations of odor or of the sugar reward are used. Furthermore, we show that Synapsin is selectively required for larval short-term memory. Thus, without Synapsin *Drosophila* larvae can learn and remember, but Synapsin is required to form memories that match in strength to event salience—in particular to a high saliency of odors, of rewards, or the salient recency of an event. We further show that the residual memory scores upon a lack of Synapsin are not further decreased by an additional lack of the Sap47 protein. In combination with mass spectrometry data showing an up-regulated phosphorylation of Synapsin in the larval nervous system upon a lack of Sap47, this is suggestive of a functional interdependence of Synapsin and Sap47.

One of the brain's more fascinating features is that it allows the organism to learn and to remember. Learning and memory fine-tune the way an animal can act in its environment, e.g., in the search for food. Using odor–sugar reward associative learning in larval *Drosophila* as a study case, we investigate the role of the Synapsin protein in learning and memory (Scherer et al. 2003; Neuser et al. 2005; Saumweber et al. 2011; for reviews, see Gerber et al. 2009; Diegelmann et al. 2013).

Synapsins constitute a family of evolutionarily conserved phosphoproteins. They are associated with the cytoplasmic side of synaptic vesicles and tether vesicles to the cytoskeleton, thus forming a reserve pool (Greengard et al. 1993; Hosaka et al. 1999; Südhof, 2004; Hilfiker et al. 2005). In *Drosophila*, Synapsin is encoded by only one gene and is expressed in most if not all neurons of both the larval and adult nervous system (coding gene: *syn*, CG 3985: Klagges et al. 1996; Michels et al. 2005). Both adult and larval *Drosophila* lacking Synapsin show associative memory scores that are reduced by about half as compared with wild-type animals, as do animals upon an RNAi-mediated knockdown of Synapsin (adult odor–punishment memory: Godenschwege et al. 2004; Knapek et al. 2010; Niewalda et al. 2015; Walkinshaw et al. 2015; larval odor–reward memory: Michels et al. 2005, 2011). Corresponding phenotypes in learning and memory tasks have been reported throughout the animal kingdom, including man (Silva et al. 1996; Garcia et al. 2004; Südhof, 2004; Gitler et al. 2008; Greco et al. 2013).

In both larval and adult *Drosophila*, animals lacking Synapsin exhibit normal task-relevant sensorimotor performance as indicated by normal naïve responsiveness to odors, sugar–reward, and electric shock punishment as well as normal odor detection after training-like exposure to these stimuli (Michels et al. 2005; Knapek et al. 2010; Niewalda et al. 2015). The memory impairment of Synapsin null mutant larvae can be rescued by acute transgenic Synapsin expression locally in the mushroom bodies but not by expression in the projection neurons that convey olfactory input to them (Michels et al. 2011) (acute mushroom body expression rescues memory scores for the association of odors and electric shock punishment in adult *Drosophila*, too: Niewalda et al. 2015). Thus, a Synapsin-dependent odor–reward memory trace in larval *Drosophila* arguably is local to the mushroom bodies, a third-order “cortical” brain region of the insects (Tomer et al. 2010).

Notably, phosphorylation seems to be important in the mode of operation of Synapsin (Angers et al. 2002; Fiumara et al. 2004; Giachello et al. 2010; Michels et al. 2011; Sadanandappa et al. 2013). The working hypothesis for Synapsin function is that the type I adenylate cyclase (coding gene: *rut*, CG9533) detects a coincidence of odor-induced activity in mushroom body neurons on the one hand, and of an internal aminergic reinforcement signal on the other hand, such that the cAMP–PKA cascade is activated in an odor-specific subset of mushroom body neurons (Tomchik and Davis 2009; Gervasi et al. 2010). Arguably, Synapsin is one of the target proteins of PKA (Fiumara et al. 2004; Michels et al. 2011) such that upon phosphorylation of Synapsin its affinity to the cytoskeleton is reduced and reserve-pool vesicles can be recruited. Thus, when the trained odor is encountered thereafter, more synaptic vesicles will be available for release (Shupliakov et al. 2011). It should be noted that Synapsin harbors consensus motifs for other kinases as well (Nuwal et al. 2011; Sadanandappa et al. 2013; Niewalda et al. 2015). Therefore, the net effect of odor–reward learning on the balance between reserve-pool and releasable vesicles and on synaptic transmission is difficult to predict. In any event, the modulated output from the mushroom body neurons is thought to code the learned valence of the odor and thus is the basis for learned olfactory behavior (Séjourné et al. 2011; Plaçais et al. 2013; Aso et al. 2014a,b; Menzel 2014). In this sense, Synapsin operates during learning to establish a memory trace, i.e. an altered functional state of an odor-specific set of mushroom body output synapses.

Based on electrophysiology as well as behavioral analyses, it has been suggested that the regulation of synaptic transmission via Synapsin may be particularly important to maintain high levels of transmission upon continuous, heavy demand (Godenschwege et al. 2004; Bykhovskaia 2011; Vasin et al. 2014). Regarding our odor–reward learning paradigm, we therefore predicted that Synapsin is particularly critical for forming memories of highly salient events. To put this to the test, we parametrically vary odor as well as sugar salience (both affect memory scores in wild-type larvae: Schipanski et al. 2008; Mishra et al. 2013) and ask whether Synapsin is selectively involved in forming stronger memories for high concentrations of odor and/or of the sugar reward.

In odor–punishment memory of adult *Drosophila*, Synapsin is specifically required for short-term but not longer-term memory (Knapek et al. 2010). Considering the above-mentioned working hypothesis for Synapsin function this is conceivably because the changes in the phosphorylation pattern of Synapsin are transient. Regarding the present larval odor–sugar learning task, we therefore decided to test memory at various retention intervals to see whether Synapsin is selectively necessary for short-term and/or longer-term memory.

As mentioned above, memory scores in Synapsin null mutants typically are not abolished but reduced to about half, a finding that we confirm in the present study. We have observed the same partial memory defect in null mutants of another presynaptic protein, namely Sap47 (Saumweber et al. 2011). The **s**ynapse **a**ssociated **p**rotein of **47** kDa (coding gene: *sap47*, CG 8884) has been identified by a monoclonal antibody from the Wuerzburg hybridoma library (Reichmuth et al. 1995; Funk et al. 2004; Hofbauer et al. 2009). Within this study, we ask whether Synapsin and Sap47 work in different, parallel pathways, or in series. To this end, we test for additive defects in memory of Synapsin/Sap47 double mutants. The rational for this is that no additivity should be observed if Synapsin and Sap47 operate in series, i.e., within the same process.

Last, for adult *Drosophila* several phosphorylation sites of Synapsin have been identified by mass spectrometry (Nuwal et al. 2011; Niewalda et al. 2015). We therefore decided to determine the phosphorylation status of Synapsin in larvae as well. In addition, we look for differences in the pattern of Synapsin phosphorylation between wild-type and Sap47 null mutant larvae, as such differences would be suggestive of a functional interdependence of Synapsin and Sap47.

## Results

### Genetic and molecular status

**Figure 1.**
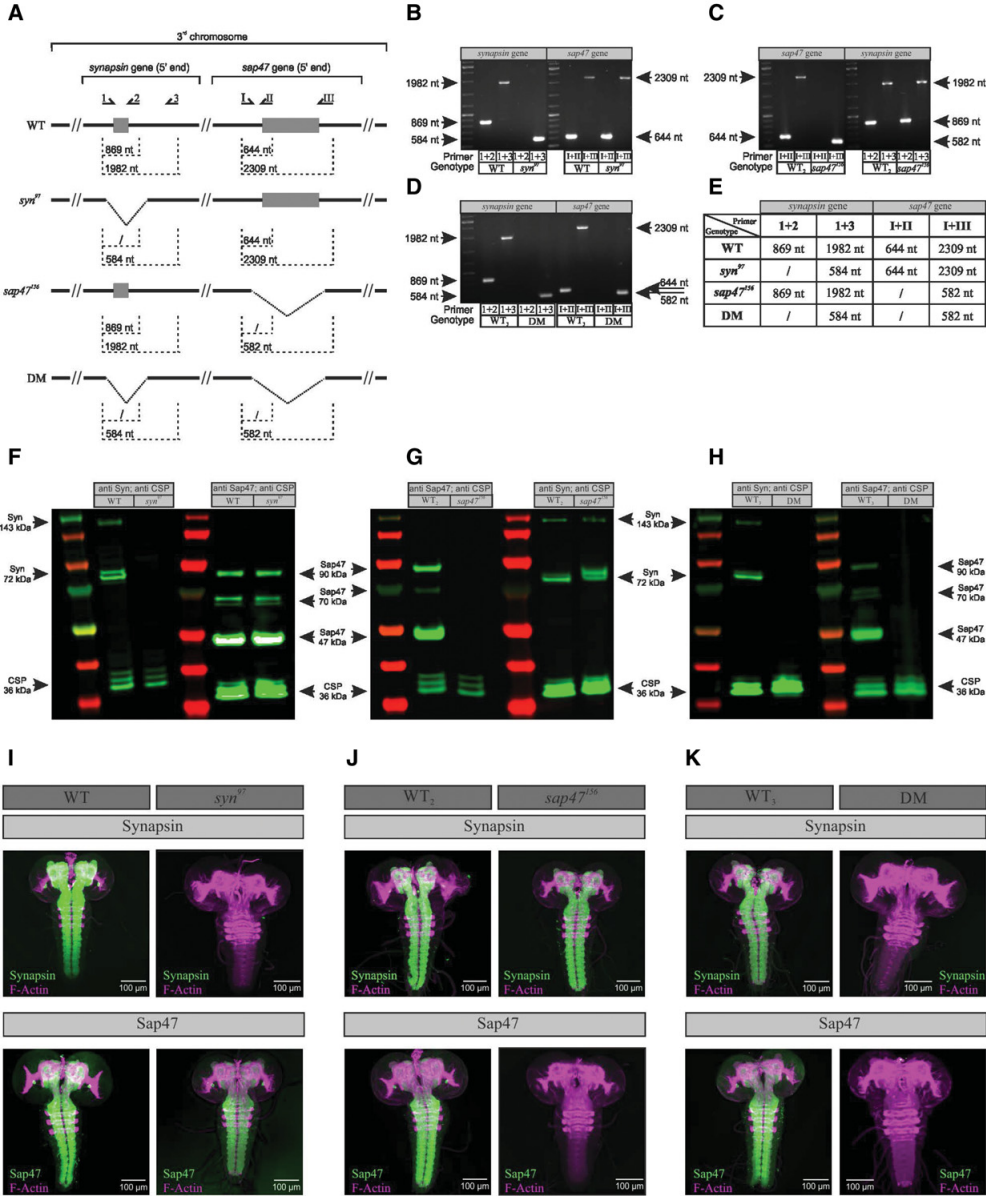
Validation of genetic and molecular status. (*A*) Overview of the primer binding sites and the expected PCR products with regard to the *synapsin* and the *sap47* gene. The primer binding sites were upstream (primer 1 for *syn* and primer I for *sap47*), within (primer 2 for *syn* and II for *sap47*), or downstream (primer 3 for *syn* and III for *sap47*) of the respective deletion. (*B*–*E*) The *syn*^*97*^, *sap47*^*156*^, and the double-mutant strains carry the reported deletions. Results of the PCR show the expected products for all genotypes used in this study. (*F*–*H*) Western blot of larval brains. (*F*) In the wild-type WT strain the anti-Synapsin antibody SYNORF1 detects expected Synapsin bands, namely a double band at 72 kDa and a weaker band at 143 kDa, whereas the *syn*^*97*^ mutant is lacking all Synapsin protein (Godenschwege et al. 2004; Michels et al. 2005). The anti-Sap47 antibody nc46 labels expected Sap47 bands at 47 kDa, 70 kDa, and 90 kDa in both the wild-type WT strain and the *syn*^*97*^ mutant, showing that the Sap47 protein is intact. (*G*) The wild-type WT_2_ strain shows expected Sap47 bands, while in the *sap47*^*156*^ mutant strain no Sap47 protein is expressed. The Synapsin protein is present in both wild-type WT_2_ strain and the *sap47*^*156*^ mutant. We note an additional anti-Synapsin band at ∼72 kDa in the *sap47*^*156*^ mutant. (*H*) The wild-type WT_3_ strain shows expected Synapsin and Sap47 bands, while the *syn*^*97*^/*sap47*^*156*^ double mutant is lacking both the Synapsin and the Sap47 proteins. In all blots, the first and fourth lane from the *left* shows the marker ladder. As loading control we used CSP as labeled by the ab49 antibody showing bands at 36 kDa for all blots (Zinsmaier et al. 1990, 1994). (*I*–*K*) Whole mounts of larval brains and ventral nerve cord. (*I*) The *left* two tiles show whole-mount preparations from wild-type WT larvae, stained with anti F-actin for orientation plus anti-Synapsin (*upper left* tile) or plus anti-Sap47 (*lower left* tile) (magenta: anti F-actin, green: anti Synapsin or anti Sap47, respectively; the individual channels are shown in Supplemental Fig. S10). Note that both the Synapsin and the Sap47 protein, if expressed, are expressed throughout the larval nervous system. The *right* panel of tiles shows the same as the left panel, but for the *syn*^*97*^ mutant, which lacks the Synapsin protein but expresses Sap47. (*J*) Same as in (*I*), showing that the wild-type WT_2_ strain expresses both Synapsin and Sap47, while the *sap47*^*156*^ mutant expresses Synapsin but lacks the Sap47 protein. (*K*) Same as in (*I*,*J*), showing that the wild-type WT_3_ strain expresses both Synapsin and Sap47, while the double mutant (DM) lacks both these proteins. All antibodies used are the same as in *F*,*G*. Scale bar: 100 µm.

Using PCR, Western blotting and whole-mount brain preparations we tested all the strains used in this study for the status of the *synapsin* and *sap47* genes and the expression of their Synapsin and Sap47 protein products in the larva (Fig. 1).

The *syn*^*97*^ mutant strain carries the reported 1.4 kb deletion in the *synapsin* gene, removing part of the promote region, exon 1, and a small part of the first intron; consequentially, it lacks all Synapsin protein (Godenschwege et al. 2004; Michels et al. 2005). In the wild-type (WT) strain, we confirm expected Synapsin protein isoforms between 70 and 80 kDa and a weaker and variable band at 143 kDa (Klagges et al. 1996). The *sap47* gene and the Sap47 protein isoforms, as expected, are intact in the *syn*^*97*^ mutant strain.

The *sap47*^*156*^mutant strain carries the reported 1.7 kb deletion, which removes part of the promoter region, the first exon, and a small part of the first intron; it therefore is not expressing any Sap47 protein (Funk et al. 2004; Saumweber et al. 2011). In the WT_2_ strain, we confirm the expected major Sap47 band at ∼47 kDa (this band can sometimes be discerned as a double band, Funk et al. 2004) a group of weaker bands at ∼70 kDa, as well as a higher band at ∼90 kDa. As expected the *synapsin* gene and the Synapsin protein are intact in the *sap47*^*156*^mutant strain. We note that in the *sap47*^*156*^mutant strain an additional band for Synapsin can be discerned at ∼72 kDa (compare the two rightmost lanes of Fig. 1G).

The *syn*^*97*^/*sap47*^*156*^ double-mutant strain carries the reported deletions in the *synapsin* as well as in the *sap47* gene (see above) and thus it is expressing neither the Synapsin nor the Sap47 protein. In the WT_3_ strain we verified genomic status and protein expression as described above.

Whole-mount brain preparations confirm these conclusions (Fig. 1I–K).

### Odor–sugar memory in *syn*^*97*^ mutants is impaired only for higher odor concentrations

**Figure 2.**
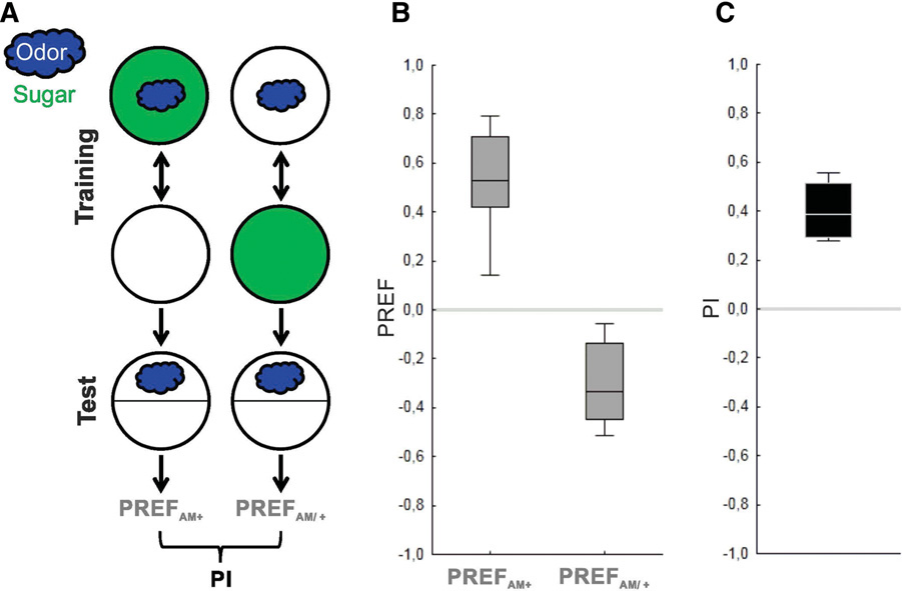
The associative learning paradigm. (*A*) Sketch of the learning paradigm for larval associative reward learning in its one-odor version, (*B*) the resulting odor preferences, and (*C*) associative performance indices of wild-type WT larvae. Using a Petri-dish assay plate (circles), groups of ∼30 larvae were trained with either of two reciprocal training regimen, namely either with a paired or an unpaired protocol. For paired training the odor, e.g., n-amyl acetate (AM) (blue cloud) is presented together with the sugar reward (green fill of circle). In the subsequent test, odor preference is calculated as the number of larvae on the odor side minus the number of larvae on the other side divided by the total number of larvae (PREF_AM+_). A second group of 30 larvae is trained reciprocally, that is by presenting odor and reward separately and the preference score is determined as described (PREF_AM/+_). The associative performance indices (PIs) are calculated as the difference between PREF_AM+_ and PREF_AM/+_, divided by 2, and are thus a measure of associative memory within the boundaries of −1 to 1. Positive PI values indicate appetitive associative memory, zero PI values indicate no learning effect, and negative values imply aversive associative memory. Box plots represent the median as the middle line, 25% and 75% quantiles as box boundaries, as well as 10% and 90% quantiles as whiskers, respectively.

Using an established odor–sugar associative learning paradigm in wild-type WT larvae (Fig. 2; Scherer et al. 2003; Neuser et al. 2005; Saumweber et al. 2011), an initial attempt to reproduce the reported *syn*^*97*^ mutant defect in odor–sugar memory failed (Supplemental Fig. S1A,B). Comparing our procedures to the published ones, however, revealed that we had used a substantially lower concentration of *n*-amylacetate (AM) (a 1:1600 dilution rather than the 1:50 dilution of AM used in both Michels et al. 2005, 2011). Subsequently using the higher concentration of AM (1:50), the published defect of the *syn*^*97*^ mutant was reproduced (Supplemental Fig. S1C,D). This prompted us to investigate systematically whether the *syn*^*97*^ mutant phenotype depends on odor concentration.

**Figure 3.**
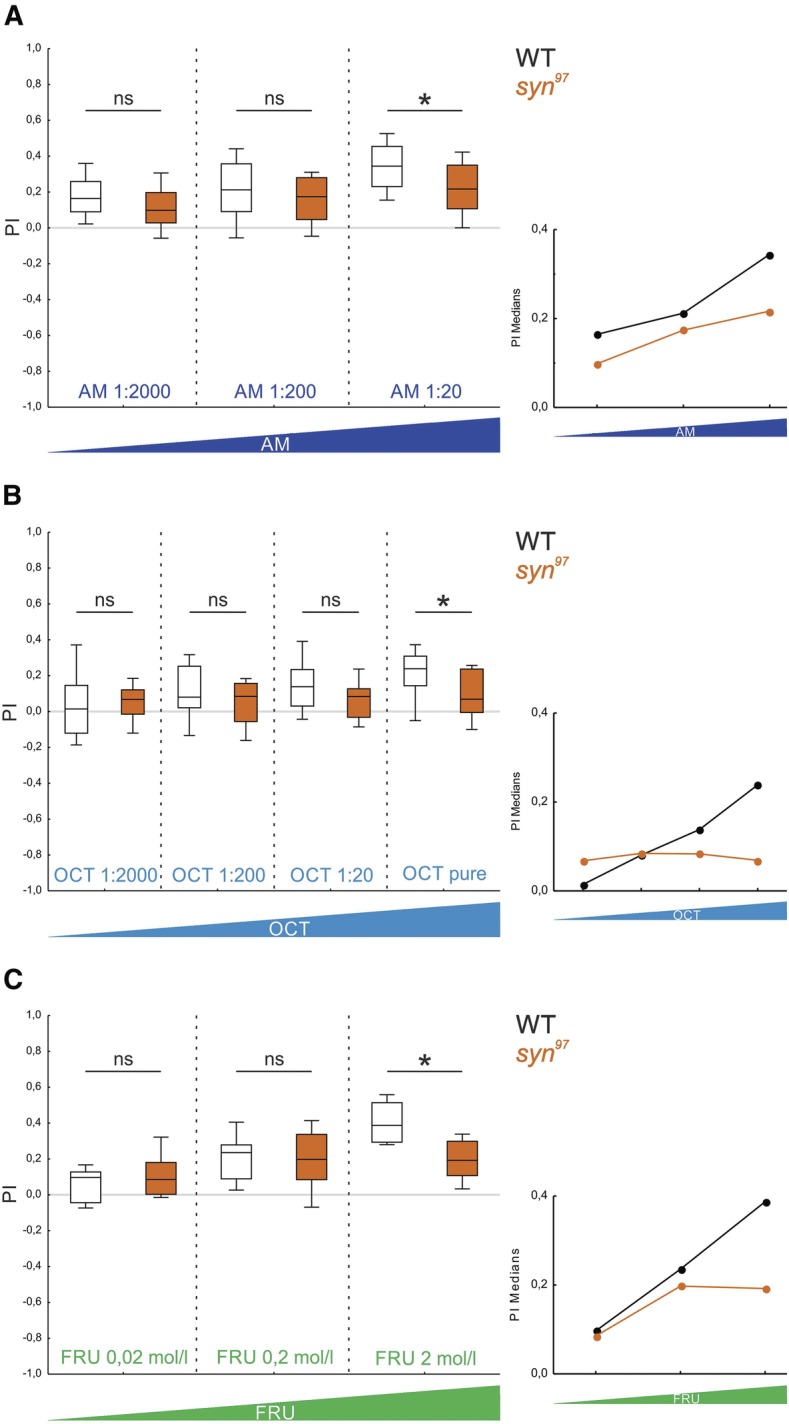
Odor–sugar memory in *syn*^*97*^ mutants is impaired selectively for high odor or sugar concentrations. (*A*) As higher concentrations of the odor AM were used (dark blue), defects in odor–sugar memory of the *syn*^*97*^ mutant strain become apparent (the inset shows the median PIs plotted across AM concentrations). White fill of the box plots is used for the wild-type WT strain, orange fill for the *syn*^*97*^ mutant strain. All displayed data were gathered in parallel. The underlying PREF scores are documented in Supplemental Figure S2. ns indicates *P* > 0.05/3, and (*) *P* < 0.05/3 in MWU tests. Other details as in Figure 2. (*B*) Same as in *A*, for OCT as odor. The underlying PREF scores are documented in Supplemental Figure S3. ns indicates *P* > 0.05/4, and (*) *P* < 0.05/4 in MWU tests (from *left* to *right*: *U* = 312, 293, 251, 277.5; *N* = 27, 27, 28, 28, 27, 27, 32, 32). Comparison within a given strain and across concentrations yields *P* < 0.05/2 at *H* = 16.16 for the wild-type WT strain and *P* > 0.05/2 at *H* = 1.10 for the *syn*^*97*^ mutant strain in KW tests (df = 3 in both cases). Other details as in Figure 2. (*C*) To examine whether the odor–sugar memory scores of the *syn*^*97*^ mutant are also dependent on the sugar concentration, three different fructose (FRU) concentrations were used. Only the highest fructose concentration (2 mol/L) revealed a *syn*^*97*^ mutant phenotype while for the other concentrations the memory scores of the wild-type WT and the mutant were at approximately the same level (the inset shows the median PIs plotted across fructose concentrations). All displayed data were gathered in parallel. The underlying PREF scores are documented in Supplemental Figure S4. ns indicates *P* > 0.05/3, and (*) *P* < 0.05/3 in MWU tests. Other details as in Figure 2. Regarding the wild-type WT, these results are qualitatively in line with Mishra et al. (2013) concerning odor concentration as well as with Neuser et al. (2005) and Schipanski et al. (2008) concerning sugar concentration, despite some variations in wild-type genotype and paradigm.

Using six experimental groups handled in parallel, we used three different odor concentrations, in either the wild-type WT or *syn*^*97*^ mutant larvae (1:2000, 1:200, 1:20 dilutions of AM). The defect in odor–sugar memory of the *syn*^*97*^ mutant indeed was observed for the highest but not for the two lower concentrations of AM (Fig. 3A; Supplemental Fig. S2; 1:2000: *P* > 0.05/3; 1:200: *P* > 0.05/3; 1:20: *P* < 0.05/3; *U* = 207, 306, 213; *N* = 24, 24, 27, 27, 27, 27). Specifically, in the *syn*^*97*^ mutant associative performance indices remained at a statistically uniform low level across the range of tested concentrations (*P* > 0.05/2; H = 7.22; df = 2; sample sizes as above). In contrast, the scores of wild-type WT larvae were higher for higher concentrations of AM (*P* < 0.05/2; H = 14.16; df = 2; sample sizes as above). Strikingly, the same pattern of results was found for another odor, OCT (Fig. 3B, Supplemental Fig. S3). It thus appears that in the *syn*^*97*^ mutant, different from the wild-type WT (Fig. 3A,B; Mishra et al. 2013), memory strength cannot be properly adjusted to be higher for higher odor concentrations. This made us wonder whether a similar effect would be seen if stronger memories are established on the basis of a stronger reward (Schipanski et al. 2008). In other words, is Synapsin required when a particularly strong memory needs to be established when particularly salient cues are to be associated?

### Odor–sugar memory in *syn*^*97*^ mutants is impaired only for higher sugar concentrations

We used three different concentrations of the fructose reward (FRU; 0.02, 0.2, 2 mol/L) (and AM as odor at the 1:20 dilution which is permissive for detecting the defect of the *syn*^*97*^ mutant). It turns out that only at the highest FRU concentration a *syn*^*97*^ mutant phenotype was detectable, while for the other concentrations memory scores of the wild-type WT and the mutant were at the approximately same level (Fig. 3C, Supplemental Fig. S4; 0.02 mol/L: *P* > 0.05/3; 0.2 mol/L: *P* > 0.05/3; 2 mol/L: *P* < 0.05/3; *U* = 65, 81, 26; *N* = 12, 12, 13, 13, 15, 15). Across sugar concentrations we observed statistically uniform scores for the *syn*^*97*^ mutant (*P* > 0.05/2; H = 3.98; df = 2; sample size as above), while associative performance indices of wild-type WT were higher for higher sugar concentrations (*P* < 0.05/2; H = 25.40; df = 2; sample size as above). Thus, the wild-type WT but not the *syn*^*97*^ mutant can adjust memory strength to be higher when higher sugar concentrations are used during training.

Taken together, in the absence of Synapsin *Drosophila* larvae can form odor–sugar memories, yet Synapsin is required in order to adjust memory strength to a higher salience of odors or of the reward for establishing stronger memories.

### *syn*^*97*^ mutants are selectively impaired in short-term memory

**Figure 4.**
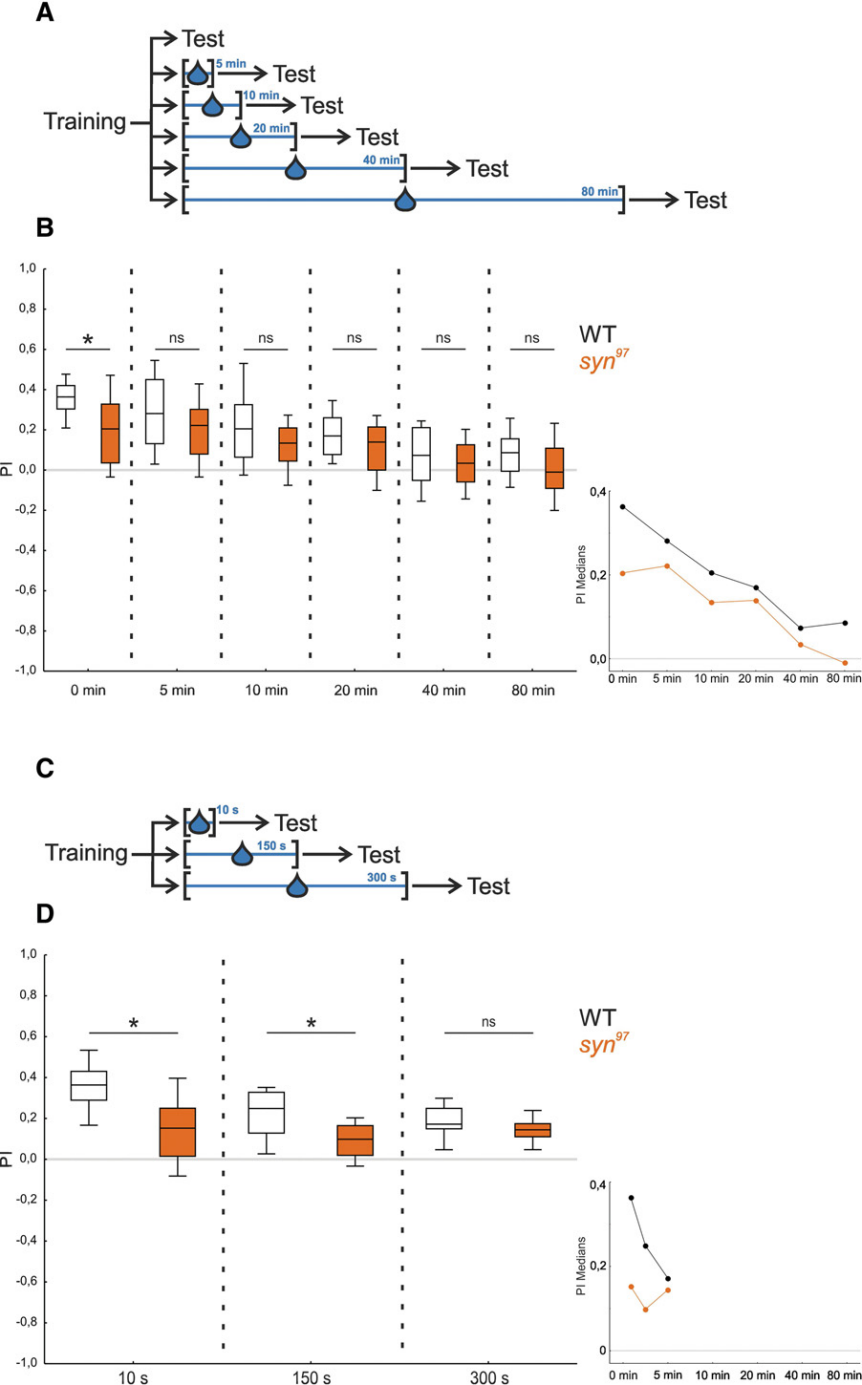
*syn*^*97*^ mutants are selectively impaired in short-term memory. To investigate memory over time, the *syn*^*97*^ mutant and the wild-type WT were tested at different time points after training. (*A*) The wild-type WT strain and the *syn*^*97*^ mutant strain were tested at one of six different time points after training. During the time interval between training and test the larvae were placed into a drop of water in all cases, except in the case when the larvae were tested immediately after training (0 min). (*B*) Only immediately after training (test at 0 min) lower associative memory scores for the *syn*^*97*^ mutant than the wild-type WT were found, while for all later testing time points no difference in memory scores was observed (inset, showing the median PIs plotted across time intervals). All displayed data were gathered in parallel. The underlying PREF scores are documented in Supplemental Figure S5A. (*) indicates *P* < 0.05/6, and ns indicates *P* > 0.05/6 in MWU tests. Other details as in Figure 2. (*C*) The experiment was repeated such that all experimental groups were placed into a water droplet, and only the duration of the retention period was varied. (*D*) Both at 10 sec and at 150 sec after training the *syn*^*97*^ mutant larvae showed an impairment in memory that was gone after 300 sec/5 min (inset, showing the median PIs plotted across time intervals). Therefore, the decrease in memory early after training is related to a time-dependent process. All displayed data were gathered in parallel. The underlying PREF scores are documented in Supplemental Figure S5B. (*) indicates *P* < 0.05/3, and ns indicates *P* > 0.05/3 in MWU tests. Other details as in Figure 2.

Memory typically is strong immediately after an event, and degrades over time. Is Synapsin required for the early “extra” memory component that supports high levels of learned behavior shortly after training? We tested separate groups of wild-type WT and *syn*^*97*^ mutant larvae at one of six different time points after training: either immediately after training (0 min) or after retention intervals ranging from 5 to 80 min (Fig. 4A). The stimuli were chosen to be conducive to detecting a phenotype (AM diluted 1:20; FRU 2 mol/L). In order to create a situation during the retention interval that was different from both the training and the test situation, the larvae were placed onto a plain plastic dish into a drop of water for the indicated time intervals.

We found that the *syn*^*97*^ mutant showed a defect in memory only immediately after training (0 min) but not for any of the later time points (Fig. 4B; Supplemental Fig. S5A; 0 min: *P* < 0.05/6; 5, 10, 20, 40, and 80 min: *P* > 0.05/6; *U* = 232.5, 301, 283.5, 242.5, 275, 232.5; *N* = 31, 31, 28, 28, 28, 28, 25, 25, 26, 26, 26, 26). For both genotypes we observed a decay of associative performance indices over time (*P* < 0.05/2 in both cases; H = 53.71, 30.54 for wild-type WT and *syn*^*97*^; df = 5 in both cases; sample sizes as above).

Given the experimental design (Fig. 4A), it remained unclear whether the requirement of Synapsin reflects a merely time-dependent process, and/or whether the placement into the water droplet is an amnesic treatment, such that Synapsin-dependent memory is erased in the wild-type WT strain (no such confound is present for the corresponding finding of Knapek et al. 2010 in adult *Drosophila*). We therefore repeated the experiment such that all experimental groups were placed into a water droplet and only the duration of the retention period was varied (either 10, 150, or 300 sec: Fig. 4C). We found that the *syn*^*97*^ mutant showed an impairment in memory after both 10 and 150 sec whereas, in confirmation of the above results (Fig. 4B), after 300 sec no difference in memory was detectable between the *syn*^*97*^ mutant and the wild-type WT (Fig. 4D, Supplemental Fig. S5B; 10 and 150 sec: *P* < 0.05/3; 300 sec *P* > 0.05/3; *U* = 34, 51, 76; *N* = 15 for all groups). Within this narrow time range we detected a decay of memory scores for the wild-type WT (Fig. 4D; WT: *P* < 0.05/2; H = 12.5; df = 2; sample sizes as above) while memory scores of the *syn*^*97*^ mutant remained effectively stable (Fig. 4D; *syn*^*97*^: *P* > 0.05/2; H = 1.75; df = 2; sample sizes as above).

Thus, Synapsin is required to form memories that support high levels of learned behavior shortly after training.

### *syn*^*97*^/*sap47*^*156*^ double mutants show no additive impairment in odor–sugar memory

**Figure 5.**
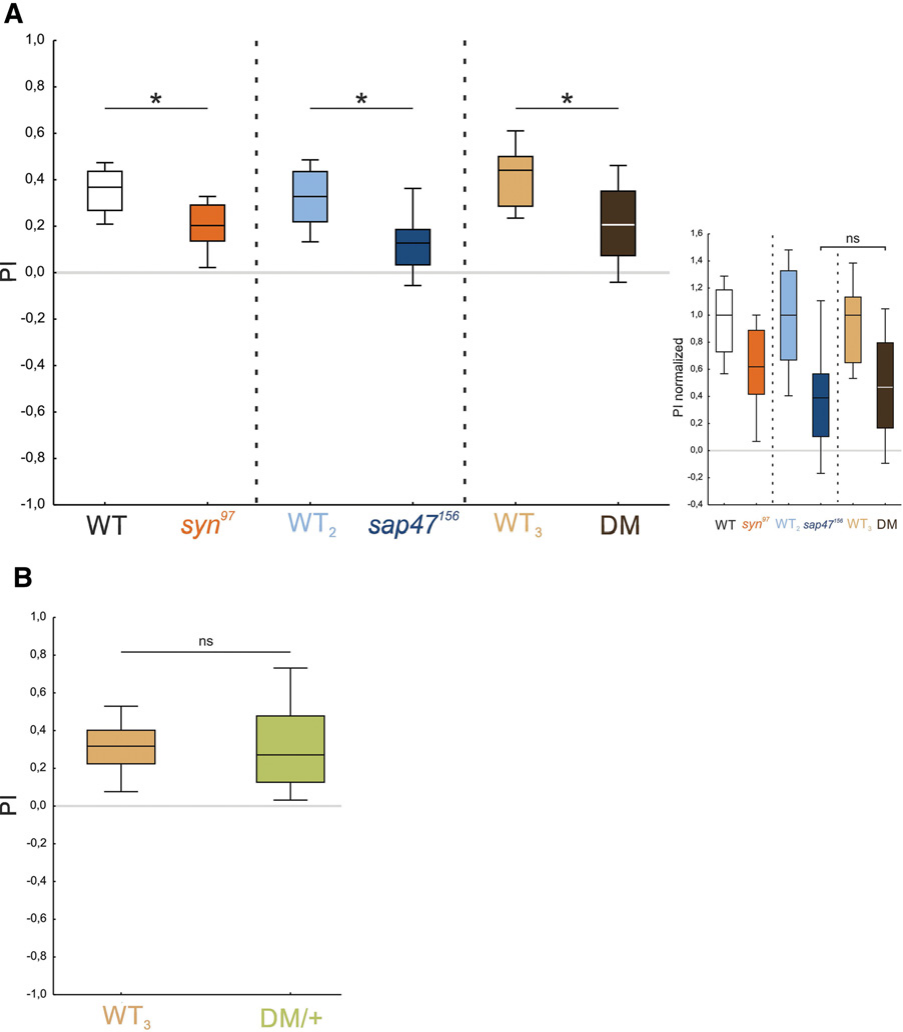
The memory impairments in the *syn*^*97*^ and the *sap47*^*156*^ mutants are not additive. (*A*) Memory was compared between the *syn*^*97*^ mutant, the *sap47*^*156*^ mutant, and the *syn*^*97*^/*sap47*^*156*^ double mutant (DM) to investigate whether there is an additive memory defect for the double mutant. All mutants are significantly impaired in memory compared with their respective wild-type. The memory scores of the *syn*^*97*^ mutant were reduced by ∼40% compared with the wild-type WT; the *sap47*^*156*^ mutant showed a reduction in memory of ∼60% compared with the wild-type WT_2_. The DM revealed an impairment in memory of ∼60% in comparison to the wild-type WT_3_ (inset, showing the normalized PIs, ns indicates *P* > 0.05 in a MWU test). Hence no additive effect was detected. The color of the fill of the box plots is used to indicate genotype. All displayed data were gathered in parallel. The underlying PREF scores are documented in Supplemental Figure S6A. (*) indicates *P* < 0.05/3 in MWU tests. Other details as in Figure 2. (*B*) Heterozygous *syn*^*97*^/*sap47*^*156*^ double mutants (DM/+) showed no impairment in memory compared with the wild-type WT_3_. The underlying PREF scores are documented in Supplemental Figure S6B. ns indicates *P* > 0.05 in MWU test. Other details as in Figure 2.

Given the conspicuous residual 50% of associative memory in the *syn*^*97*^ mutant, we wondered what the genetic determinants for this remaining capacity are. Specifically, we wondered whether the defect in the *syn*^*97*^ mutant would be additive with the decrease in associative memory scores, likewise of about 50%, observed in the *sap47*^*156*^ mutant (Saumweber et al. 2011). An additive defect in memory would result if the Synapsin and Sap47 proteins were acting in parallel to support memory. In contrast, a lack of additivity implies an absence of evidence for such parallel organization, and rather suggests that they are acting in series, within the same process. We therefore tested for associative memory in the *syn*^*97*^ mutant, the *sap47*^*156*^ mutant, and a *syn*^*97*^/*sap47*^*156*^ double mutant (DM), as well as their corresponding wild-type strains (WT, WT_2_, WT_3_, respectively, see Materials and Methods for nomenclature). Based on the previous data, this experiment featured AM at a 1:20 dilution, and 2 mol/L FRU as reward. All three mutants showed a significant and ∼40%–60% impairment in associative function compared with their respective wild-type (Fig. 5A; Supplemental Fig. S6A; *P* < 0.05/3 in all cases; *U* = 104, 103.5, 117; *N* = 22, 22, 24, 24, 25, 25). When memory scores are normalized to the respective wild-type performance, scores of the *sap47*^*156*^ mutant, which is the one showing the stronger defect, and the DM are indistinguishable (Fig. 5A inset; *P* > 0.05; *U* = 261; *N* = 24, 25). With due caveats in mind (see Discussion), such a lack of additivity upon the lack of both Synapsin and Sap47 is suggestive of both proteins not working in parallel, but rather within the same process to confer associative memory. Our results from the analytical chemistry of the Synapsin and Sap47 proteins are consistent with such functional interdependence (see next section).

We note that heterozygous *syn*^*97*^/*sap47*^*156*^ double mutants (DM/+: heterozygous for both the *syn*^*97*^ mutation and the *sap47*^*156*^ mutation; Supplemental Fig. S8) showed no impairment in memory compared to the wild-type WT_3_ (Fig. 5B; Supplemental Fig. S6B; *P* > 0.05; *U* = 568; *N* = 36, 36). Accordingly, neither the *syn*^*97*^ nor the *sap47*^*156*^ mutation are dominant in their effect on memory, meaning single functional alleles of the *synapsin* and *sap47* genes are sufficient to ensure proper associative function.

### Synapsin phosphorylation is altered in *sap47*^*156*^ mutants

**Figure 6.**
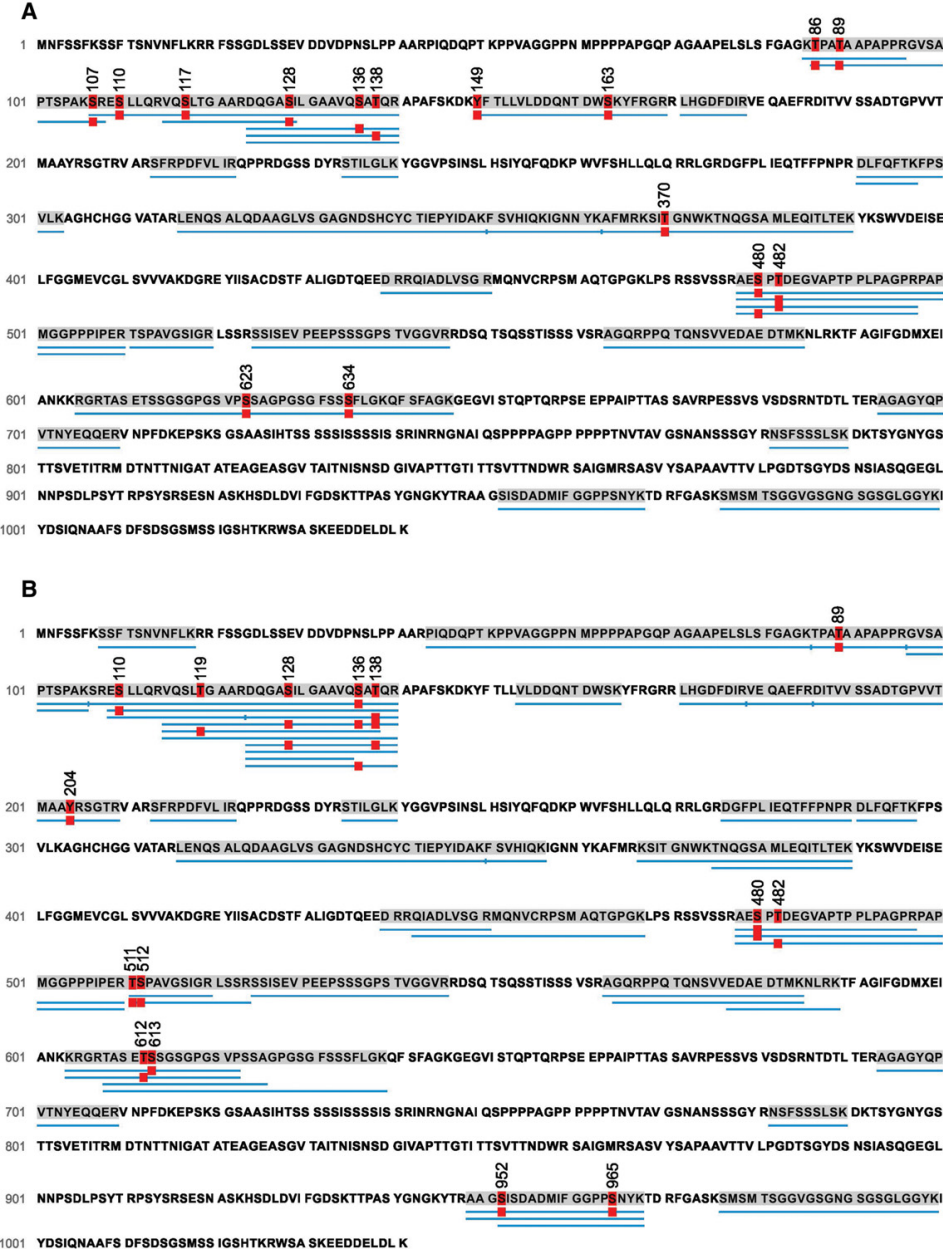
The pattern of Synapsin phosphorylation is altered in *sap47*^*156*^ mutants. Phosphorylation sites of Synapsin in experimentally naive wild-type WT_2_ (*A*) and *sap47*^*156*^ mutant larvae (*B*). Thirteen LC-MS/MS runs were performed to analyze the phosphorylation status across the Synapsin protein in both genotypes. The number of times a phosphopeptide or its corresponding nonphosphorylated counterpart was detected is indicated as counts in Table 1. (*A*) We identified 15 phosphosites of Synapsin in the wild-type WT_2_ and (*B*) 15 phosphorylated sites of Synapsin in the *sap47*^*156*^ mutant larvae. Blue bars below the sequence indicate the peptides identified as peptide-spectra matches (PSM) using the PEAKS de novo sequencing algorithm. The red “P” boxes indicate phosphorylation (*P* < 0.005). As an example how to read this display and Table 1, in the wild-type WT_2_ all peptides covering amino acids 478–497 were found to be phosphorylated at either S480 or S482, but in no case were both or neither of these two found to be phosphorylated. Table 1 then shows that a phosphorylated S480 site was found for 8 out of 15 peptides, while for S482 phosphorylation was observed for the remaining 7 peptides.

Considering a possible interdependence of Synapsin and Sap47 function (see previous section), and given the additional Synapsin band in Western blots of *sap47*^*156*^mutant larvae (Fig. 1G, two rightmost lanes) as well as the functional significance of the phosphorylation of Synapsin in general (see Introduction), we decided to compare the phosphorylation of the Synapsin protein from larval brains of *sap47*^*156*^ mutants to the corresponding wild-type WT_2_. Using mass spectrometry (LC-MS/MS) we achieved coverage of 38% of the Synapsin protein in WT_2_ and of 47% in the *sap47*^*156*^mutant strain; within the covered regions, we ascertained 15 different phosphorylated sites of the Synapsin protein from experimentally naïve wild-type WT_2_ larvae (Fig. 6A; Table 1). Of note, Synapsin was always phosphorylated at a central motif, namely at either S480 or S482; no case was observed with phosphorylation lacking at both these sites, or with phosphorylation present at both these sites. The same applies in the *sap47*^*156*^ mutant (Fig. 6B)—with the striking difference that it is almost always only S480 that is phosphorylated (Table 1).

**Table 1.**
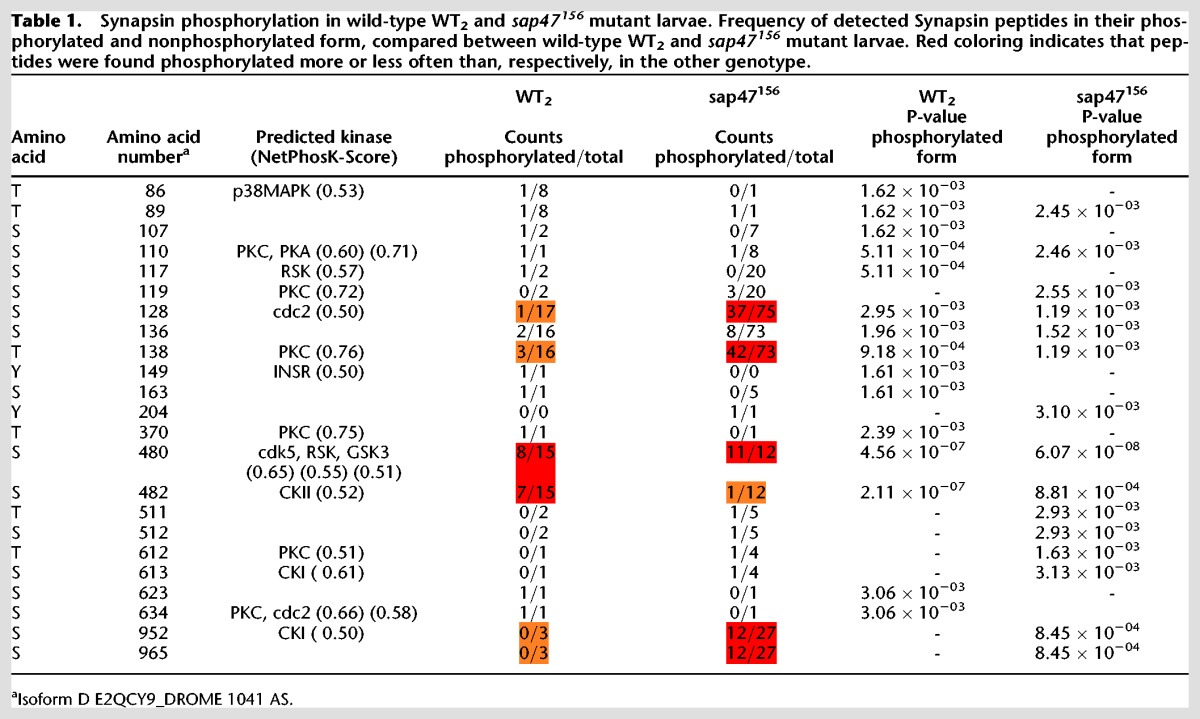
Synapsin phosphorylation in wild-type WT_2_ and *sap47*^*156*^ mutant larvae. Frequency of detected Synapsin peptides in their phosphorylated and nonphosphorylated form, compared between wild-type WT_2_ and *sap47*^*156*^ mutant larvae. Red coloring indicates that peptides were found phosphorylated more or less often than, respectively, in the other genotype.

Particularly frequent instances of Synapsin phosphorylation were observed in *sap47*^*156*^mutant larvae not only at S480, but also at a more amino-terminal motif, at S128 and T138 (Table 1). Regarding this amino-terminal motif, we find a higher number of differently phosphorylated kinds of Synapsin in the *sap47*^*156*^mutant than in the wild-type WT_2_ (Fig. 6A,B). Last, we would like to mention that in a carboxy-terminal region a double-phosphorylation at S952 and S965 was found in the *sap47*^*156*^ mutant, but not in the wild-type WT_2_.

These alterations of Synapsin phosphorylation in the *sap47*^*156*^ mutant are suggestive of a functional interplay between Synapsin and Sap47.

## Discussion

*Drosophila* larvae lacking Synapsin can form and remember odor–reward associations, but as we show Synapsin is required to profit from a high salience of odors or from a high salience of the reward in order to establish strong memories (Fig. 3). Likewise, the early “extra” memory component that supports high levels of learned behavior shortly after training, that is memory for saliently recent events, is Synapsin-dependent (Fig. 4).

### Synapsin is required for short- but not longer-term memory

Our observation that Synapsin is required specifically for short- but not longer-term odor–reward memory in larval *Drosophila* matches what Knapek et al. (2010) found for odor–punishment memories in adult *Drosophila*. Given the requirement of Synapsin for regulating the balance between reserve-pool and releasable vesicles in a phosphorylation-dependent way (see Introduction), this seems plausible. The training-induced changes in the phosphorylation pattern of Synapsin are likely transient, such that the initial balance between reserve-pool vesicles and releasable vesicles is relatively quickly resumed. In effect, Synapsin function thus is the basis for the memory of saliently recent events.

### Synapsin boosts memory strength for highly salient events

According to the working hypothesis for odor–reward learning in *Drosophila* (see Introduction and Supplemental Fig. S9), it is straightforward to understand why strong rewards lead to strong odor–reward memories. A stronger reward would more strongly activate a dopaminergic reward signal, leading to a stronger activation of inter alia the AC-cAMP–PKA-Synapsin pathway in those mushroom body Kenyon cells that are coincidently activated by the odor. Thus, more reserve vesicles would be recruited and a stronger memory trace established. Without Synapsin, this ability to adapt memory strength to reward strength is compromised. Certainly, the eventual net effect on synaptic strength would include the effects of other activated kinases, too (see Introduction).

At first sight it seems equally straightforward that a high odor concentration will activate the mushroom body Kenyon cells more strongly and, as in the case of a strong reward, establish a stronger memory. However, according to such a scenario one would predict equal or higher memory scores if the odor concentration is increased between training and test. This is because during the test with a higher-than-trained odor concentration the mushroom body Kenyon cells would be activated more strongly, leading to at least as strong output as with the trained odor concentration. Contradicting this prediction, memory scores were found to be less when odor concentration was increased between training and test (i.e., memory is specific for the trained odor intensity: Mishra et al. 2013; also Yarali et al. 2009). In terms of physiology, both the level of activity and the combination of activated mushroom body Kenyon cells varies, albeit slightly, with odor concentration*.* It will be interesting to see whether and which parameter set of biologically plausible mushroom body models (Luo et al. 2010, Nehrkorn et al. 2015) can account for both the high memory scores found when using a high odor concentration in training and in testing (Fig. 3A,B; Mishra et al. 2013, loc. cit. Fig. 2), as well as for the decrease in memory scores when the odor concentration is increased between training and test (Mishra et al. 2013, loc. cit. Fig. 3). The circuit motif suggested by Nehrkorn et al. (2015) in principle seems to be capable of capturing both these aspects.

In punishment learning of adult flies, event salience has been varied by introducing temporal gaps between the stimuli to be associated. This revealed both Synapsin-dependent and Synapsin-independent punishment memory components for optimally timed, highly salient, events. For suboptimally timed, less salient cases, punishment memory is Synapsin-independent (Niewalda et al. 2015). The data set from Niewalda et al. (2015) is revealing also in another respect. That is, for optimal punishment learning the odor is presented shortly before the shock (forward conditioning), yielding punishment memory scores of PI ≈ −0.6. When the sequence of odor and shock is reversed such that the odor is presented only upon the pleasantly relieving cessation of shock (backward conditioning), flies subsequently approach that odor. Such “relief” memory typically is weaker than punishment memory, yielding scores of only PI ≈ 0.2, even at an optimal backward interval (Gerber et al. 2014). Such relief memory is Synapsin-dependent. Interestingly, when a suboptimal forward conditioning interval is used, punishment memory is just as weak as relief memory after optimal backward conditioning (PI ≈ −0.2 and 0.2, respectively)—yet in the *syn*^*97*^ mutant a decrement in relief memory but not in punishment memory is observed (Niewalda et al. 2015, loc.cit. Fig. 3B,C)! Thus, the absolute level of memory does not appear to be the sole determinant for the involvement of Synapsin. Rather, the requirement of Synapsin becomes the more obvious the closer the memory process operates at its particular upper limit.

We conclude that Synapsin is required to form memories such that they match in strength to high event salience, either in relation to odor salience, reward salience, event-recency, or event-timing. This suggests that Synapsin may be required whenever a memory process operates to its particular upper limit.

### The roles of Synapsin, Sap47, and Brp for short-term memory

The present data confirm that a lack of Synapsin reduces memory scores to about half, raising the question of the nature of the residual Synapsin-independent memory. We had found earlier, and have confirmed in this study, that a lack of the Sap47 protein likewise entails a reduction of memory scores to half (Fig. 5A; Saumweber et al. 2011). Notably, the decrements in memory upon a lack of both Synapsin and Sap47 are not additive (Fig. 5A), suggesting that the residual Synapsin-independent memory is also Sap47-independent and vice versa. Clearly, one caveat regarding this suggestion is that it is based on an absence of evidence for additivity, which must not be confused with evidence of the absence of additivity. Still, the changes in phosphorylation of Synapsin upon a lack of Sap47 suggest an interdependence of the function of the two proteins (Table 1; Fig. 6). Whether the altered phosphorylation of Synapsin in particular at the amino-terminal (S128/138), central (S480/482), and/or the carboxy-terminal phospho motif (S952/965) is significant with respect to memory function remains to be investigated. Interestingly, the memory defect of mutants lacking Synapsin cannot be rescued by a Synapsin protein with mutated S22 and S549 sites (Michels et al. 2011; loc. cit. S6/S533); these sites were found to be phosphorylated in adult *Drosophila* (Niewalda et al. 2015), but unfortunately the present analysis, despite our efforts, does not yield information about their phosphorylation status in the larva. Indeed, protein mass spectrometry for larval tissue is substantially more difficult than for adult tissue, arguably because of a lower specific abundance of Synapsin relative to total protein; enrichment of the native protein by immune-precipitation with the anti-Synapsin antibody SYNORF1 was not successful. We note that the changes in Synapsin phosphorylation in mutants lacking Sap47 are a possible cause of the additional Synapsin band seen in Western blots (Fig. 1G, two rightmost lanes) (alterations in phosphorylation of a protein can result in changes of electrophoretic mobility beyond the slight mass increases generated by the additional phospho groups themselves, i.e., 79.97 Da per phospho group).

In any event, what could be the molecular basis for the residual Synapsin- as well as Sap47-independent memory? Regarding olfactory punishment learning in adult *Drosophila* Knapek et al. (2010) reported that Synapsin-independent memory is amnesia-resistant. In turn amnesia-resistant short-term memory does require the Bruchpilot protein (Brp; coding gene: *brb*, CG42344), a protein localized to the presynaptic active zones and essential for the proper formation of presynaptic dense bodies and short-term synaptic plasticity (Wagh et al. 2006; Fouquet et al. 2009, Hallermann et al. 2010, Knapek et al. 2011). While the role of Brp in larval memory has not yet been tested, a possible scenario thus is that short-term memory has two components, one that depends on Synapsin and on Sap47, but not on Brp, and which is amnesia-sensitive; and a second component that works without Synapsin and without Sap47, requires Brp, and is amnesia-resistant.

## Materials and Methods

### Flies and rearing conditions

We used third-instar feeding stage larvae aged 5 d after egg laying. Flies were kept in mass culture and maintained at 25°C, 60%–70% relative humidity, and a 12/12-h light–dark cycle. Experimenters were blind with respect to genotype and treatment condition in all cases; these were decoded only after the experiments. We used three different wild-types together with their respective null mutants: 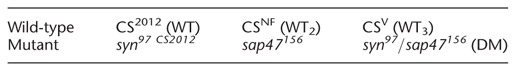


The wild-type CS^2012^ and the Synapsin mutant *syn*^*97 CS2012*^ emerged from an additional outcrossing of *syn*^*97CS*^ (Godenschwege et al. 2004; Michels et al. 2005) to wild-type CS for 13 generations. The *sap47*^*156*^ mutant strain was outcrossed to wild-type CS^NF^ for nine generations (Funk et al. 2004; Saumweber et al. 2011). Outcrossing removes marker genes introduced for mutagenesis and effectively adjusts differences in genetic background that may otherwise distort results (de Belle and Heisenberg 1996). The *syn*^*97*^/*sap47*^*156*^ double mutant was generated by V. Albertova by homologous recombination and then outcrossed to wild-type CS.

For simplicity, the wild-type CS^2012^ strain is labeled WT, the wild-type CS^NF^ strain is labeled WT_2_, and the wild-type CS^V^ strain is labeled WT_3_. The Synapsin null mutant strain *syn*^*97 CS2012*^ is labeled *syn*^*97*^ and the *syn*^*97*^/*sap47*^*156*^ double-mutant strain DM. Animals heterozygous for *syn*^*97*^ as well as for *sap47*^*156*^ are labeled DM/+.

### Single-larva PCR

To confirm the genetic status of the used strains we performed single-larva PCR in accordance with Gloor et al. (1993) (Fig. 1A,E shows the principle of primer design and the expected PCR products). The primer binding sites were upstream (first primer: 1 = *syn* primer and I = *sap47* primer), within (second primer: 2 = *syn* primer and II = *sap47* primer), or downstream (third primer: 3 = *syn* primer and III = *sap47* primer) of the respective deletion. Accordingly, the first and the second primer should yield a product only if the gene is in its wild-type condition. The first and the third primer produce a product for both the wild-type and the mutant status of the gene, which can be clearly distinguished because of their size. Specifically, the following primers were used: 
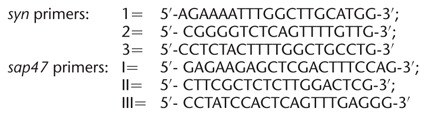


### Western blot

For homogenization and electrophoresis we used the Novex Bolt Mini Gel system (Life Technologies Carlsbad, USA). For each lane, 10 larval brains were homogenized in 10 µL homogenization buffer, containing 2.5 µL LDS sample buffer (4×), 1 µL reducing agent (10×), and 6.5 µL deionized water. The sample was heated to 70°C for 10 min and centrifuged for 30 sec before electrophoresis. For gel electrophoresis we used the Novex Bolt Mini Gel Tank. The proteins were separated in a 4%–12% Bis–Tris Plus gel at 165 V for 40 min. The proteins were transferred to nitrocellulose membrane with the iBlot Gel Transfer Device system. After the membrane was blocked with Odyssey Blocking Buffer (LI-COR, Lincoln, USA) for 1 h, it was washed three times for 10 min in 1× PBST. For the immunoreaction we used three primary monoclonal mouse antibodies. SYNORF1 was used for Synapsin detection (Klagges et al. 1996) (diluted 1:100 in PBST); for Sap47 detection nc46 (Funk et al. 2004; Hofbauer et al. 2009) (diluted 1:100 in PBST) was used; ab49 (Zinsmaier et al. 1990, 1994) (diluted 1:100 in PBST) was used for detection of the Cysteine String Protein (CSP; Arnold et al. 2004) as loading control. As secondary antibody we used IRDye 800CW goat anti-mouse lgG (LI-COR) (diluted 1:15000 in PBST). The primary antibody incubation was performed at 4°C overnight followed by three 10-min washing steps in PBST. Secondary antibody incubation at RT for 1 h was followed by three final 10-min washing steps in PBST. Detection and visualization was performed with the ODYSSEY CLx Imaging System (LI-COR).

### Immunohistochemistry

Larval brains were dissected in Ringer's solution and fixed in 3% paraformaldehyde dissolved in PBST (0.2% Triton X-100) for 1 h. After three 10-min washes in PBST (3% Triton X-100), the brains were treated in blocking solution containing 3% normal goat serum (Jackson ImmunoResearch Laboratories Inc.) in PBST for 1.5 h. Tissue was then incubated overnight with either SYNORF1 for Synapsin detection (diluted 1:10 in blocking solution) or nc46 for Sap47 detection (diluted 1:10 in blocking solution). Six 10-min washing steps in PBST were followed by incubation with a secondary rabbit anti-mouse antibody conjugated with Alexa 488 (diluted 1:200) (Invitrogen Molecular Probes). For orientation in the preparation we used overnight staining with Alexa Fluor 568 Phalloidin (diluted 1:200) (Invitrogen Molecular Probes), which visualizes filamentous actin. After final washing steps with PBST, samples were mounted in Vectashield (Vector Laboratories Inc.).

### Analysis of Synapsin phosphorylation by LC-MS/MS

Sample preparation and LC-MS/MS analysis was performed as described previously for adult *Drosophila* (Niewalda et al. 2015). In brief, brains of experimentally naïve larval *Drosophila* were dissected and lysed in 8 M urea and 1% (w/v) RapiGest SF surfactant (Waters Corp., Milford, USA) and subjected to mechanical destruction (micro glass potter and sonification). After reduction and thiomethylation of cysteine residues, proteins were digested by Trypsin (Promega, Trypsin Gold). Afterward, RapiGest detergent was removed and samples were cleaned using Empore universal resin SPE-columns (3M).

Proteome analysis was performed on a hybrid dual-pressure linear ion trap/orbitrap mass spectrometer (LTQ Orbitrap Velos Pro, Thermo Scientific) equipped with an U3000 nano-flow HPLC (Thermo Scientific). Samples were separated on a 75 μm ID, 25 cm PepMap C18-column (Dionex) applying a gradient from 2% ACN to 35% ACN in 0.1% formic acid over 220 min at 300 nL/min. The LTQ Orbitrap Velos Pro MS used exclusively CID-fragmentation with wideband activation (pseudo MS3 for neutral losses of phosphate residues) when acquiring MS/MS spectra. The spectra acquisition consisted of an orbitrap full MS scan (FTMS; resolution 60,000; m/z range 400–2000) followed by up to 15 LTQ MS/MS experiments (Linear Trap; minimum signal threshold: 500; wideband isolation; dynamic exclusion time setting: 30 sec; singly charged ions were excluded from selection, normalized collision energy: 35%; activation time: 10 msec). Raw data processing, protein identification, and phosphopeptide assignment of the high-resolution orbitrap data were performed by PEAKS Studio 7.0 (Bioinformatics Solutions). False discovery rate (FDR) was set to <1%. Phosphosites were accepted as confident for *P* < 0.005 (modified *t*-test, included in PEAKS Studio 7.0).

### Petri dish preparation, odors

As assay plates for behavioral experiments we used Petri dishes (85-mm inner diameter; Sarstedt) that were filled with 1% agarose (NEEO Ultra-Quality, Roth). We used 2 mol/L fructose (FRU; CAS: 57-48-7; Roth) as reward that was added to the agarose, unless mentioned otherwise. We used *n*-amylacetate (AM; CAS: 628-63-7; Merck) or 1-octanol as odors (OCT; CAS: 111-87-5; Merck). Odors were diluted in paraffin oil (AppliChem, 1:20 for AM and 1:20 for OCT) unless mentioned otherwise. Custom-made odor containers made of Teflon, perforated in their lids to allow odor evaporation while preventing the animals from coming into direct contact with the chemicals, were filled with 10 µL of the respective odor solution.

### Associative learning

Larvae were trained with either of two reciprocal training regimen and afterward compared for their odor preference (Fig. 2A) (for a manual, see Gerber et al. 2013): In one group of larvae AM was paired with the sugar reward (AM+), while a second group of larvae was trained with unpaired presentations of odor and reward. To equate both groups with respect to the total number of trials, in the paired group blank trials were interspersed. Then, animals from both groups were tested for their AM preference. Associative memory is indicated by a relatively higher preference for AM after AM+ training as compared with AM/+ training, and is quantified by the performance index (PI; see below).

For example, ∼30 larvae were collected from the food vial and briefly washed in tap water. Two containers loaded with AM were placed at opposing sides of an assay plate including the fructose reward (+). Immediately before training started the larvae were gently placed onto the plate using a wet brush. The assay plate was closed with a lid. The lid featured at its middle ∼15 custom-made holes (1 mm diameter) for better airflow. Then, the animals were left untreated for 5 min. Subsequently, the larvae were transferred to another assay plate, with two containers at opposing sides containing no odor (empty, EM); this time no fructose reward was included in the assay plate. This cycle of paired training (AM+) was repeated two more times, each time using fresh assay plates.

After this training, the preference of the animals for AM was recorded. Unless mentioned otherwise, the larvae were immediately placed into the middle of a fresh assay plate; that fresh testing assay plate had no fructose in it. A container with AM was placed on one side, and an empty container on the other side (EM). After 3 min the number of animals on the AM side (#_AM_), on the EM side (#_EM_) and in a 1-cm wide middle stripe (#_Middle_) was counted and the preference for AM (range −1; 1; Fig. 2B) calculated as

$$\mathrm{PREF}=({\#}_{\mathrm{AM}}-{\#}_{\mathrm{EM}})/{\#}_{\mathrm{AM}\;+\;\mathrm{EM}\;+\;\mathrm{Middle}}$$

Thus, PREF values of −1 imply full avoidance, while scores of 1 would imply full attraction.

In parallel, another set of larvae was exposed to AM without fructose on a first assay plate and then to an assay plate containing fructose and an empty container, for a total of three such cycles of unpaired training (AM/+). Then, PREF scores were determined as in Equation (1). The PREF scores of all experiments are documented in Supplemental Figures S1–S7.

For both paired and unpaired training, the sequence of trial types was reversed in every other repetition of the experiment (i.e., either as described AM+/EM and AM/EM+; or EM/AM+ and EM+/AM).

From these preference values the performance index (PI; range −1; 1; Fig. 2C) can be calculated. The PI describes the difference between the preference values after paired training (PREF_AM+_) versus after unpaired training (PREF_AM/+_) and thus indicates associative memory:

$$\mathrm{PI}=({\mathrm{PREF}}_{\mathrm{AM}+}-{\mathrm{PREF}}_{\mathrm{AM}/+})/2$$

Positive PI scores therefore indicate appetitive associative memory, while negative scores indicate aversive associative memory.

For OCT as odor, experiments were performed likewise.

## Supplementary Material

Supplemental Material
