# Supplementary material for: Synapsin is required to “boost” memory strength for highly salient events
Source: Learn Mem. 2016 Jan;23(1):9–20. doi: 10.1101/lm.039685.115 (PMC4749839; doi:10.1101/lm.039685.115)
Supplement: Supplemental Material [file supp_23_1_9__index.html]

Supplemental Material 

# Synapsin is required to “boost” memory strength for highly salient events

## Supplemental Material

**Files in this Data Supplement:**

- Fig S6.tif
- Fig S7.tif
- Fig S5.tif
- Fig S2.tiff
- Fig S10.tif
- Fig S4.tif
- Fig S3.tif
- Fig S8.tif
- Fig S1.tiff
- Fig S9.tif
- Supplemental Legends.docx
