# Supplementary figures and images for: Synapsin is required to “boost” memory strength for highly salient events
Source: Learn Mem. 2016 Jan;23(1):9–20. doi: 10.1101/lm.039685.115 (PMC4749839; doi:10.1101/lm.039685.115)

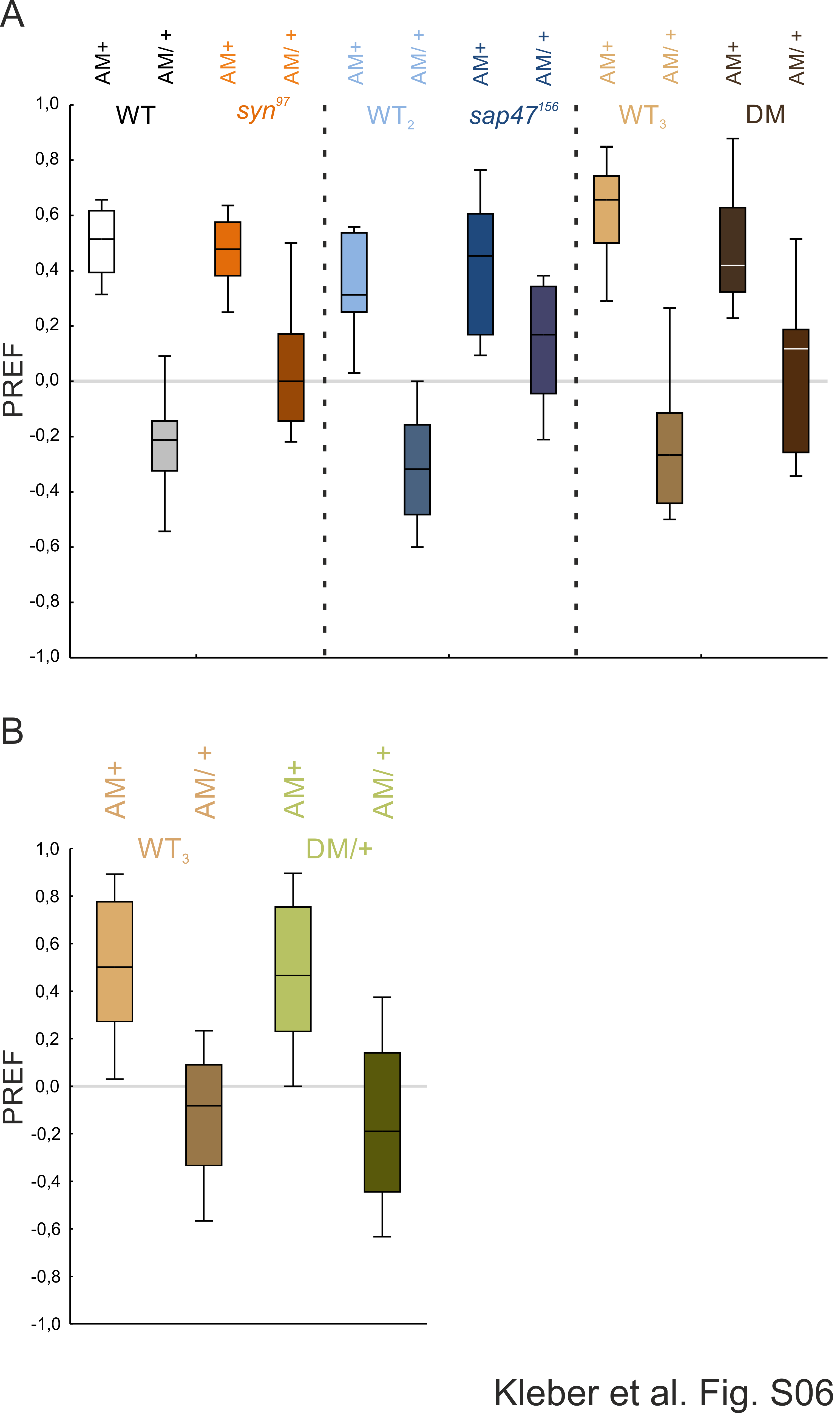

Supplement: Supplemental Material [file supp_23.1.9_Fig_S6.tif]

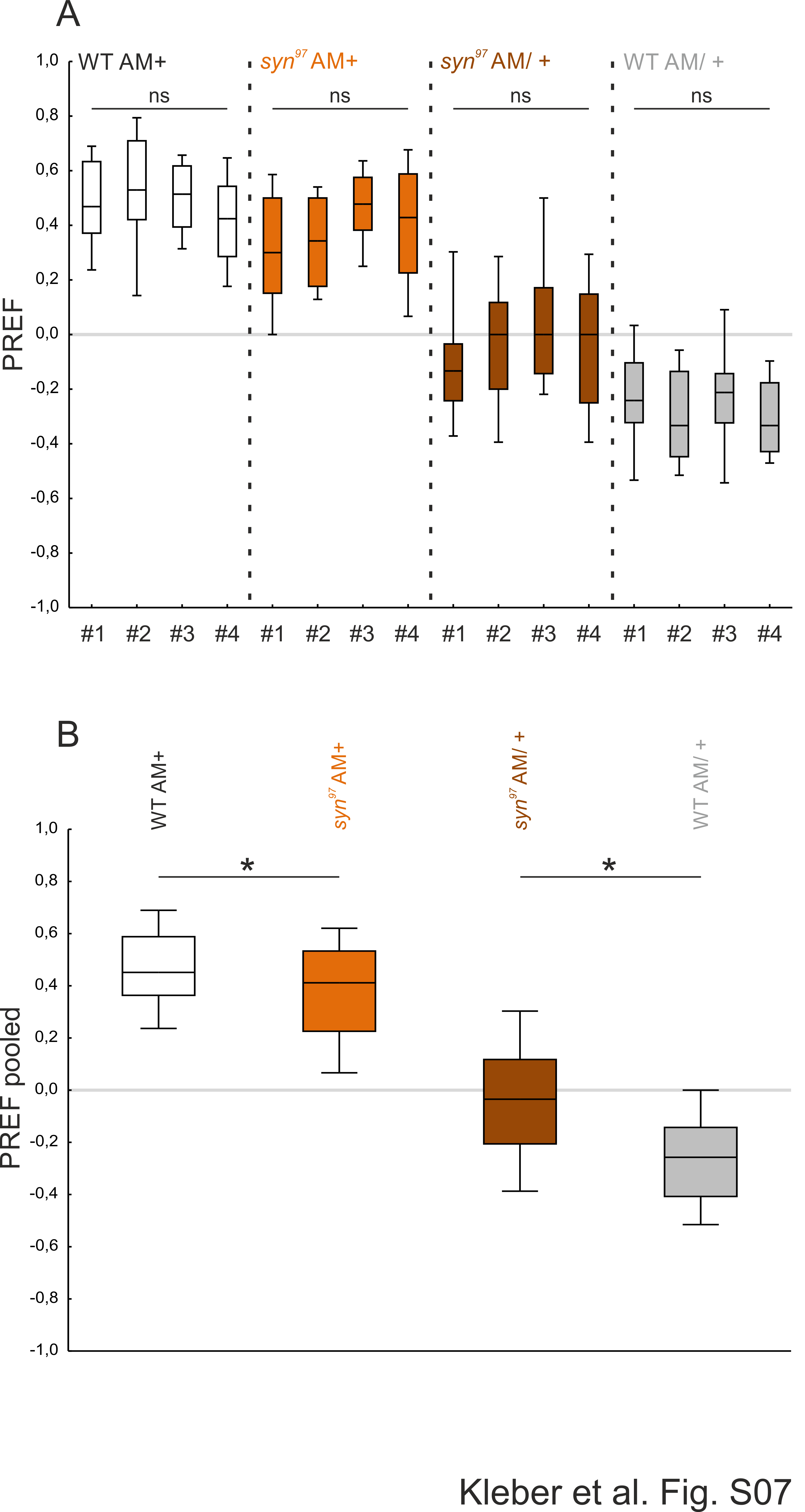

Supplement: Supplemental Material [file supp_23.1.9_Fig_S7.tif]

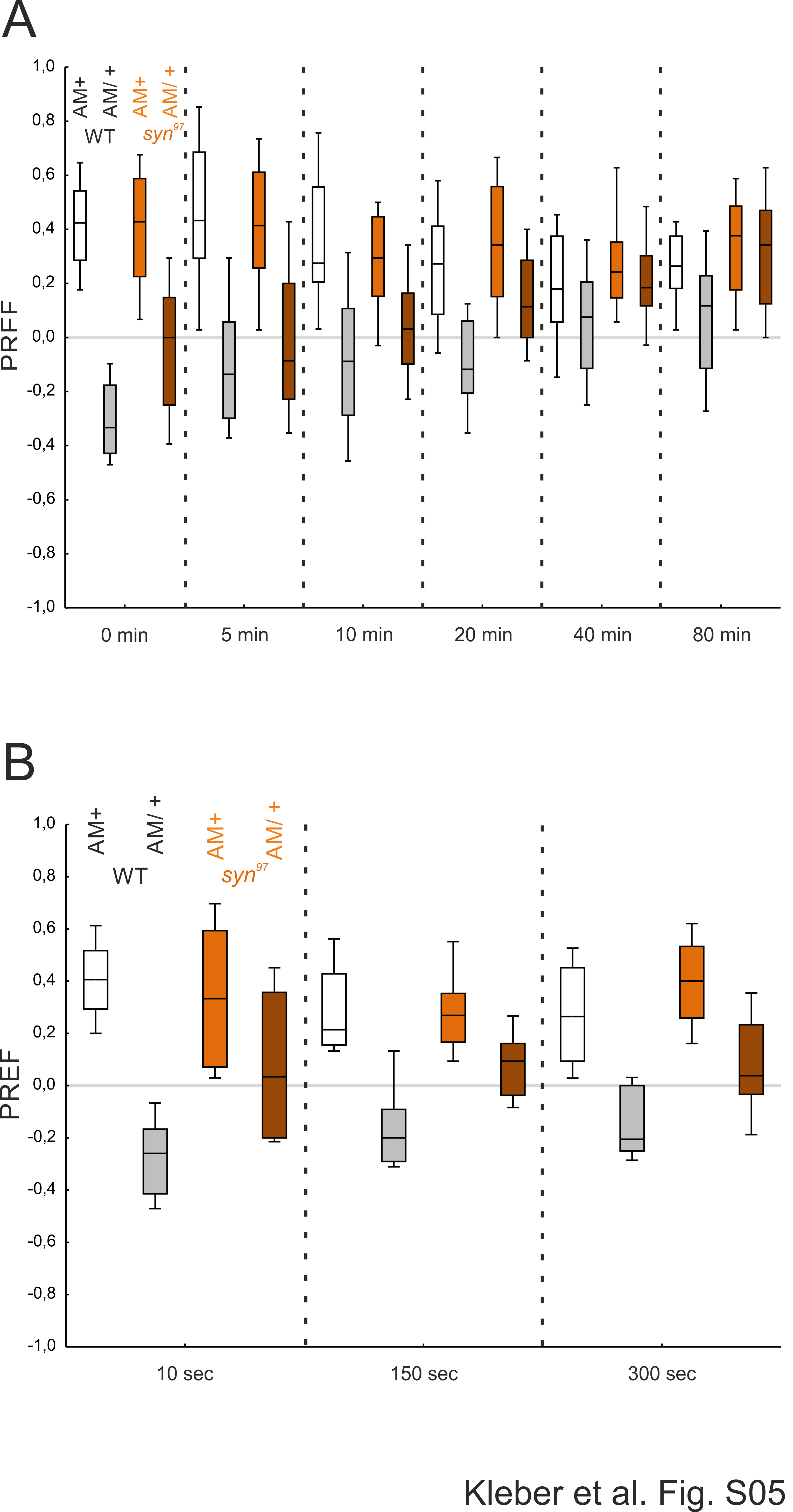

Supplement: Supplemental Material [file supp_23.1.9_Fig_S5.tif]

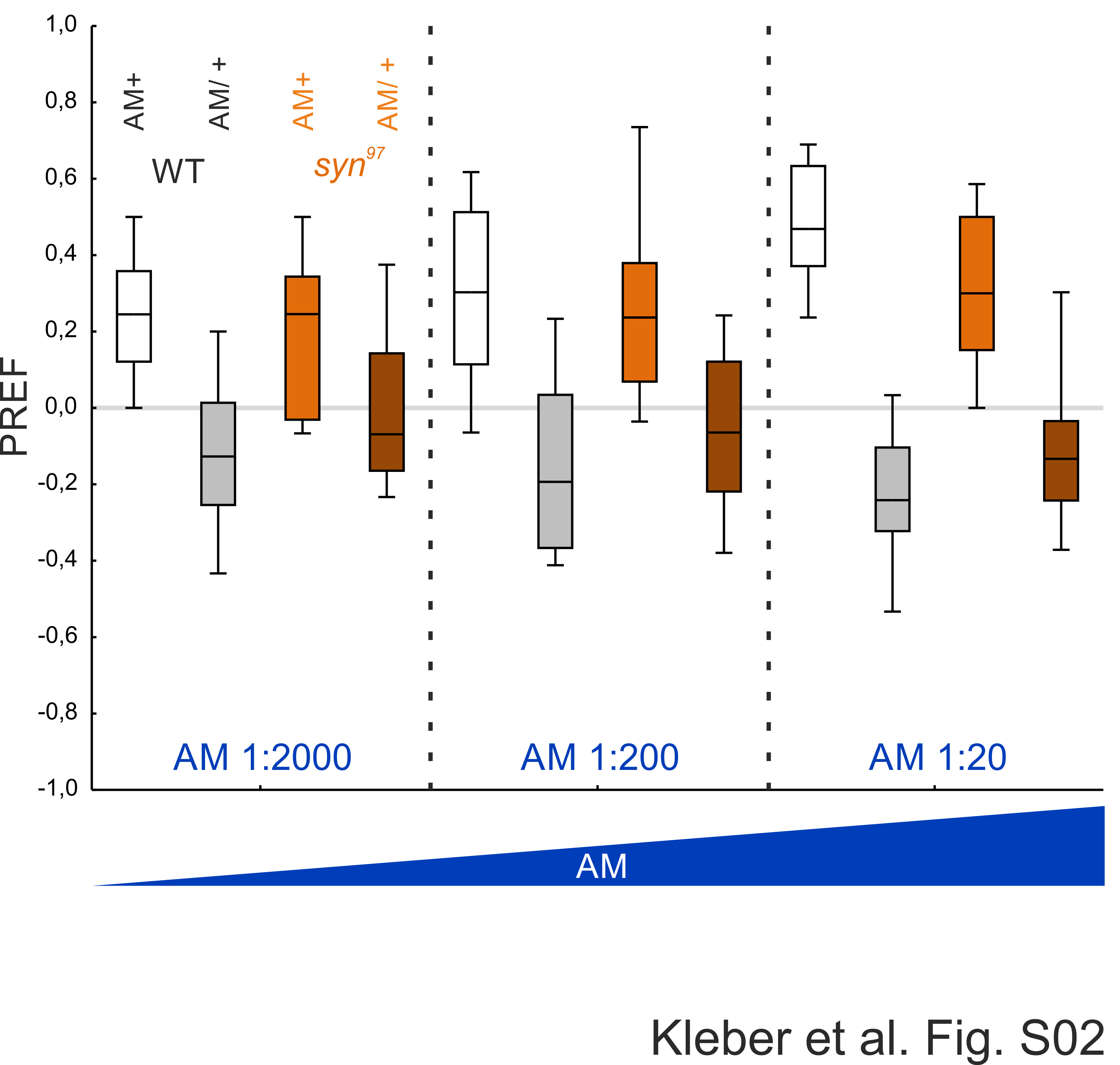

Supplement: Supplemental Material [file supp_23.1.9_Fig_S2.tif]

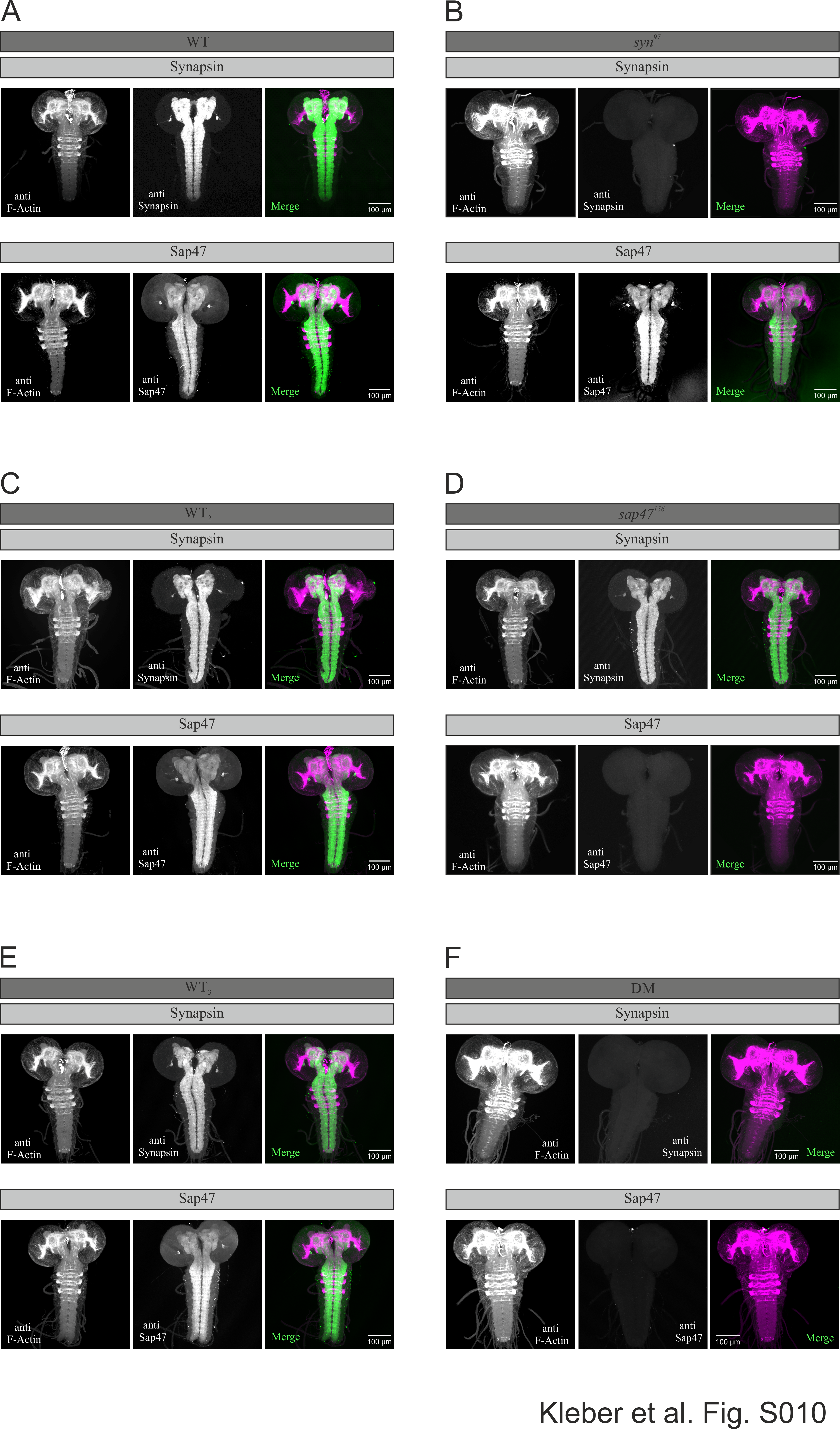

Supplement: Supplemental Material [file supp_23.1.9_Fig_S10.tif]

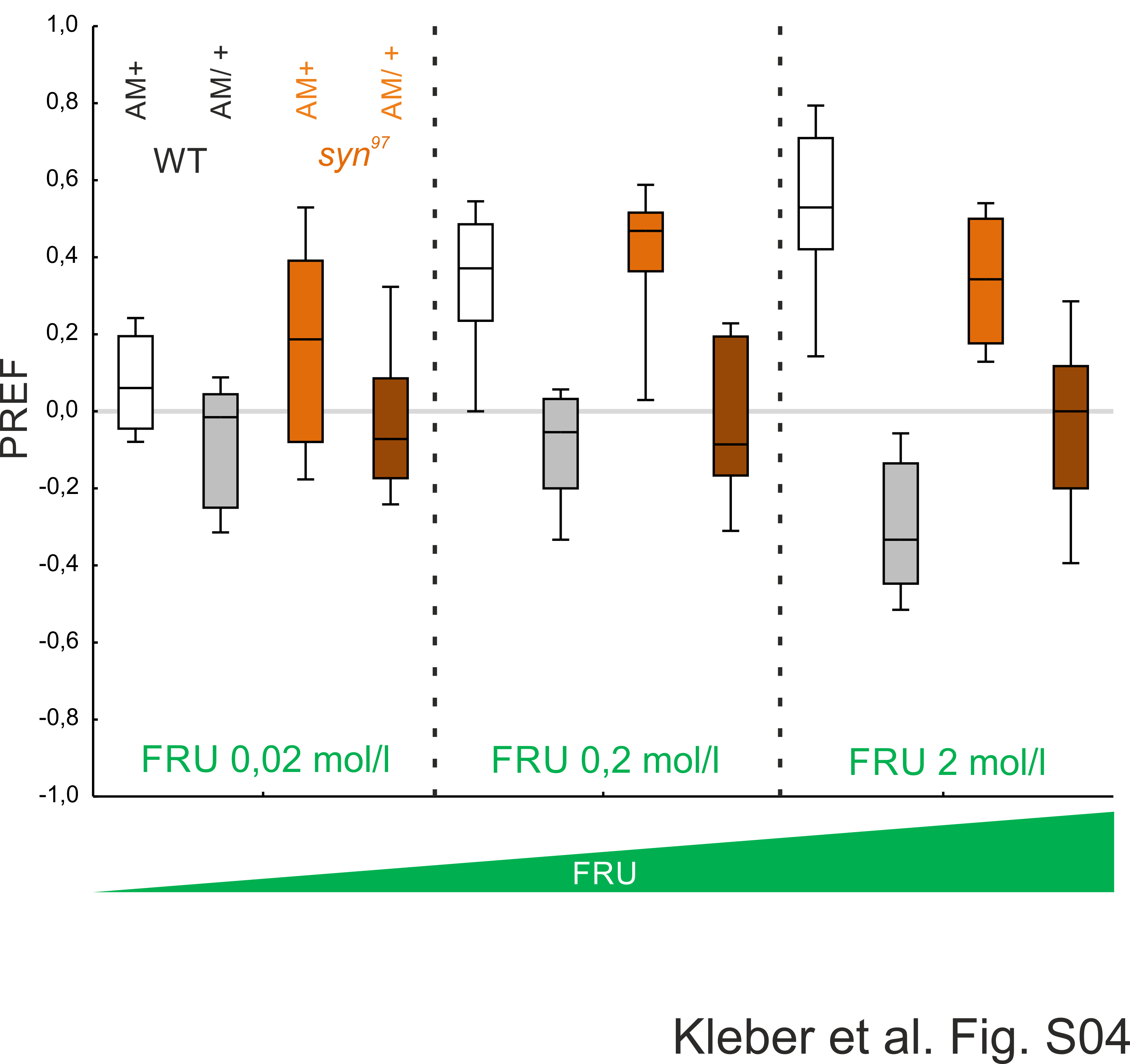

Supplement: Supplemental Material [file supp_23.1.9_Fig_S4.tif]

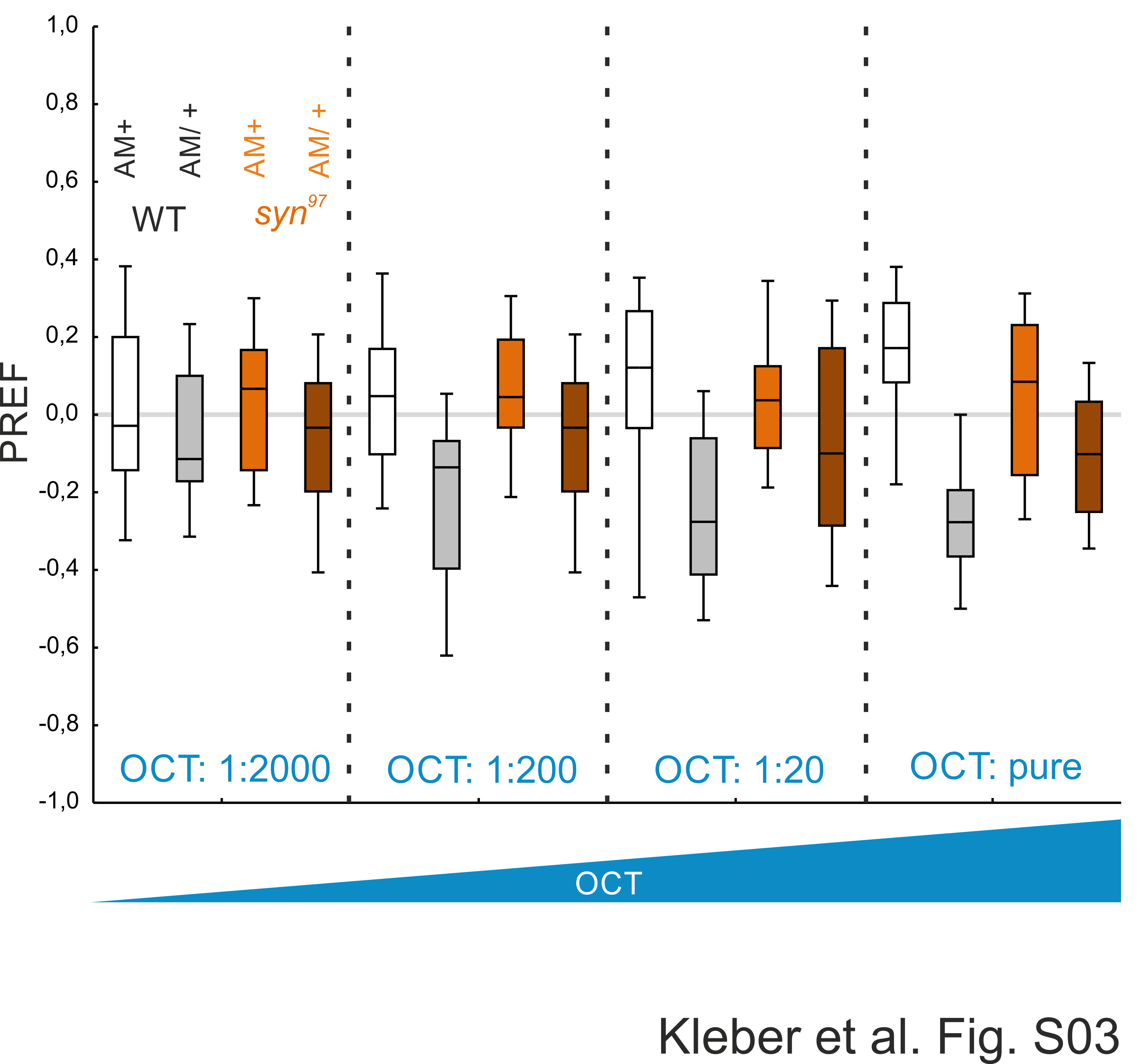

Supplement: Supplemental Material [file supp_23.1.9_Fig_S3.tif]

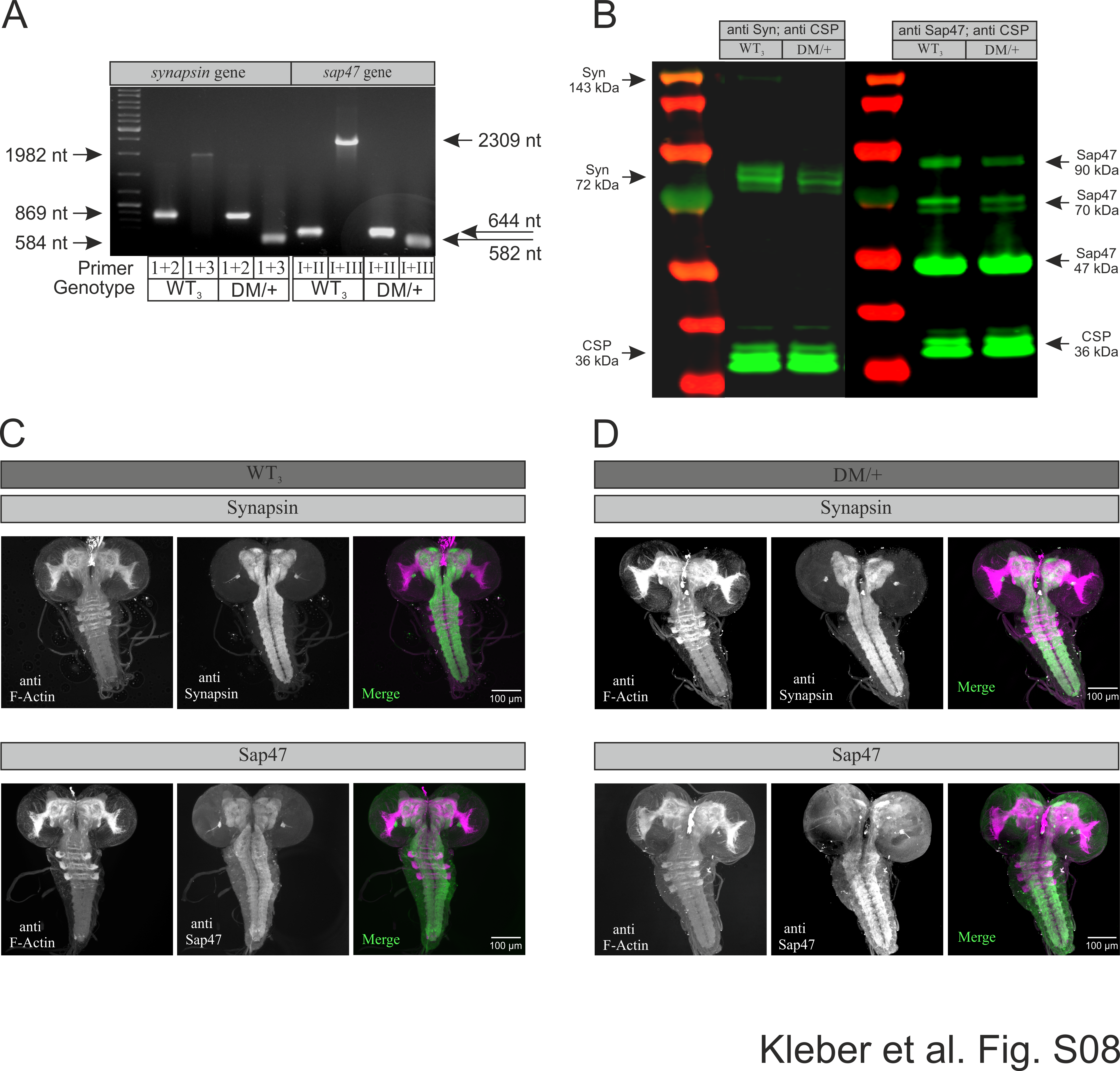

Supplement: Supplemental Material [file supp_23.1.9_Fig_S8.tif]

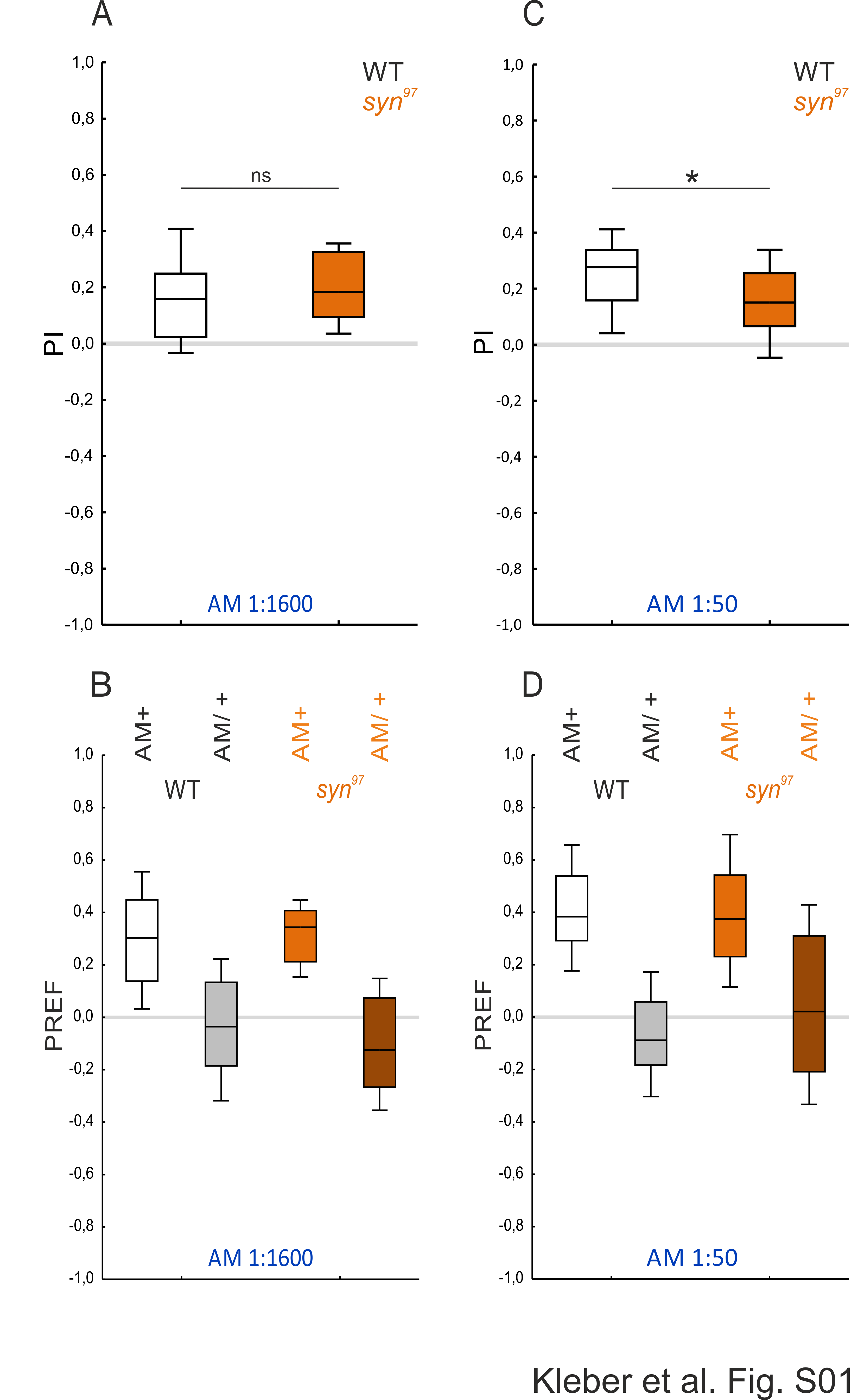

Supplement: Supplemental Material [file supp_23.1.9_Fig_S1.tif]

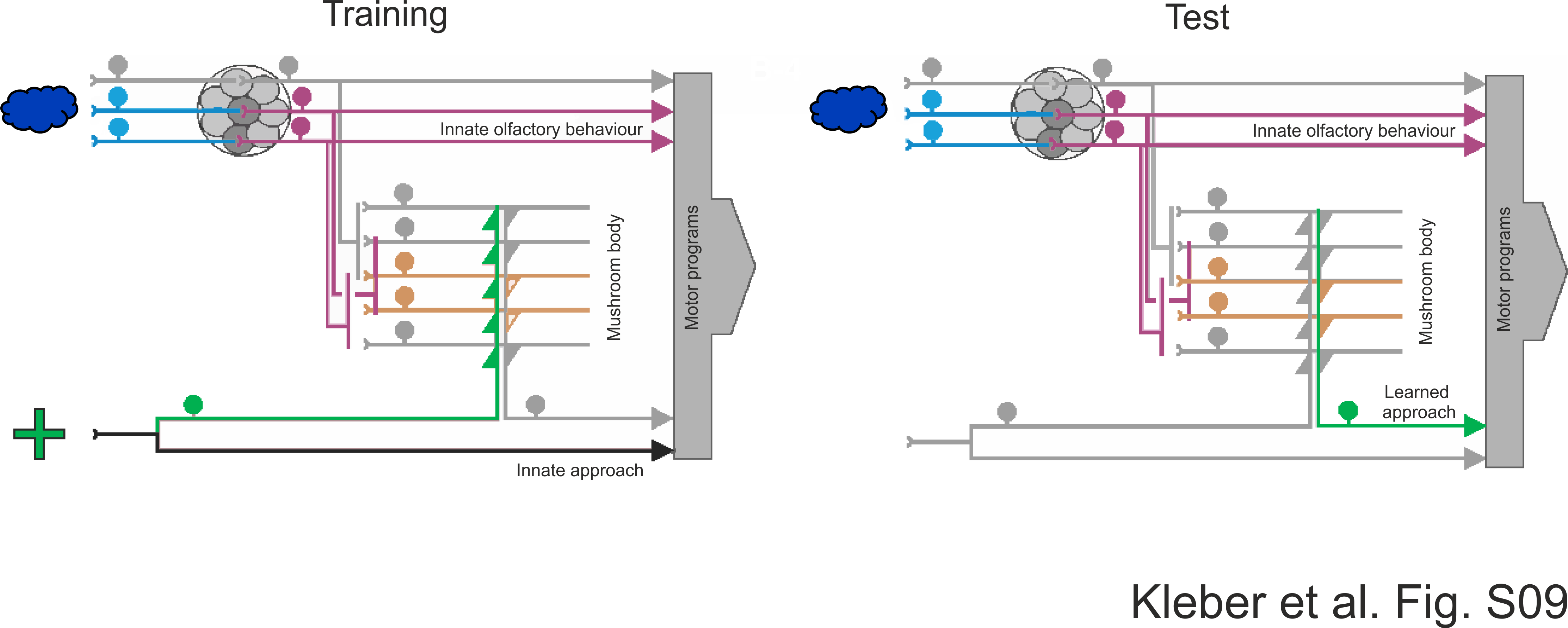

Supplement: Supplemental Material [file supp_23.1.9_Fig_S9.tif]
