# Supplementary material for: Synapsin is required to “boost” memory strength for highly salient events
Source: Learn Mem. 2016 Jan;23(1):9–20. doi: 10.1101/lm.039685.115 (PMC4749839; doi:10.1101/lm.039685.115)
Supplement: Supplemental Material [file supp_23.1.9_Supplemental_Legends.docx]

**Supplement**

**Figure S1**

Odor-sugar memory in *syn^97^* mutants is intact at low but is impaired at high odor concentration. Using the odor-sugar learning paradigm depicted in Figure 2, no memory impairment was detectable in the *syn^97^* mutant strain with a dilution of AM as odor of 1:1600 **(A) (***P*> 0.05; U= 184; N= 21, 21)**,** whereas when using a higher AM concentration in a separate set of experiments (1:50 dilution) reduced associative performance indices were observed **(C) (***P*< 0.05; U= 190; N= 21, 21**)**. White fill of the box plots is used for the wild-type WT strain, orange fill for the *syn^97^* mutant strain. **(B, D)** Corresponding PREF scores.

ns indicates *P*> 0.05, and * indicates *P*< 0.05 in MWU tests (see body text for details). Other details as in Figure 2.

**Figure S2**

Plotted are the preference scores of reciprocally trained groups of larvae from the experiment displayed in Figure 3A.

**Figure S3**

Plotted are the preference scores of reciprocally trained groups of larvae from the experiment displayed in Figure 3B.

**Figure S4**

Plotted are the preference scores of reciprocally trained groups of larvae from the experiment displayed in Figure 3C.

**Figure S5**

Plotted are the preference scores of reciprocally trained groups of larvae from the experiment displayed in Figure 4B, D.

**Figure S6**

Plotted are the preference scores of reciprocally trained groups of larvae from the experiment displayed in Figure 5A, B.

**Figure S7**

**(A)** For all experiments that were performed under the same conditions, namely with a FRU concentration of 2 mol/l and a dilution of AM of 1:20, we plotted are the preference (PREF) scores of the wild-type WT after paired training (white fill) and after unpaired training (grey fill) and the PREF of the *syn^97^* mutant after paired training (orange fill) and after unpaired training (brown fill). The corresponding experiments are displayed in Figures 3A and S2 (#1, sample size for the respective genotype and training condition N= 27, 27, 27, 27), Figures 3C and S4 (#2, N= 15 in all cases), Figures 4B and S5A (#3, N= 31 in all cases), as well as in Figures 5A and S6 (#4, N= 22 in all cases). In no case do PREF scores differ across experiments (*P*> 0.05 and df= 3 in all cases; from left to right H= 3.79; H= 4.45; H= 5.03; H= 2.86 in KKW tests, indicated by ns). Pooling across experiments **(B)** reveals statistically significant differences between the wild-type WT and the *syn^97^* mutant after both, paired training (*P*< 0.05/2; U= 3549.5; N= 95, 95) and after unpaired training (*P*< 0.05/2; U= 2219.5; N= 95, 95) (* indicates *P*< 0.05/2 in MWU tests).

**Figure S8**

**(A)** Validation of the genetic status of the double heterozygous *syn^97^/sap47^156^* mutant (DM/+) via PCR (for details see Methods section and Figure 1A, E). We note that for primer pair 1+3 as well as for primer pair I+III only the energetically favorable short fragment and not the long fragment is detected in the DM/+. **(B)** Western blots of larval brains probed for Synapsin and Sap47 of the indicated genotypes (for details see Methods section). Expected Synapsin bands at 72 kDa are present, but the Synapsin band at 142 kDa, which has been reported before to be occasionally weak or even missing (Godenschwege et al. 2004; Michels et al. 2011), is hardly detectable in the WT_3_ and is missing in the DM/+. **(C-D)** Whole mount larval brains probed for Synapsin and Sap47 of the indicated genotypes (for details see Methods section). We note that Synapsin levels appear slightly reduced in the double heterozygous *syn^97^/sap47^156^* mutant, both as judged from the Western blot (B), and the whole mounts (C, D).

**Figure S9**

Simplified circuit-level working hypothesis of the events during odor-reward training and test. Odors (blue cloud) are coded combinatorially along ascending olfactory pathways up to the mushroom body Kenyon cells (orange). Intersecting modulatory neurons convey a reward signal (green). Coincidence of odor-evoked activity in the mushroom body Kenyon cells with activity from these modulatory neurons leads to plasticity in the output synapses of the mushroom body Kenyon cells. Processing along these modified synapses then is the basis for learned avoidance upon testing (modified from Gerber et al. 2014). No learned behavior is observed if the testing odor too strongly deviates from the training odor in intensity and/or quality (Chen et al. 2011; Mishra et al. 2013), arguably because of insufficient overlap in the set of Kenyon cells relative to the trained odor.

**Figure S10**

Whole mounts of larval brains. **(A)** For the wild-type WT strain, the top row of tiles shows whole mount preparations of the larval brain hemispheres and ventral nerve cord. These are stained with anti F-actin for orientation (left tile) and with anti-Synapsin (middle tile). The rightmost tile shows the merge (magenta: anti F-actin, green: anti Synapsin). The bottom row of tiles shows the same, but for the Sap47 protein. Note that both the Synapsin and the Sap47 protein, if expressed, are expressed throughout the larval nervous system. **(B)** Same as in (A), but for the *syn^97^* mutant, which lacks the Synapsin protein but expresses Sap47. **(C, D)** Same as in (A, B), showing that the wild-type WT_2_ strain expresses both Synapsin and Sap47, while the *sap47^156^* mutant expresses Synapsin but lacks the Sap47 protein. **(E, F)** Same as in (A, B), showing that the wild-type WT_3_ strain expresses both Synapsin and Sap47, while the double mutant (DM) lacks both these proteins. All antibodies used are the same as in Figure 1F-H. Scale bar: 100 µm.
